# Cmr is a redox-responsive regulator of DosR that contributes to *M. tuberculosis* virulence

**DOI:** 10.1093/nar/gkx406

**Published:** 2017-05-08

**Authors:** Laura J. Smith, Aleksandra Bochkareva, Matthew D. Rolfe, Debbie M. Hunt, Christina Kahramanoglou, Yvonne Braun, Angela Rodgers, Alix Blockley, Stephen Coade, Kathryn E.A. Lougheed, Nor Azian Hafneh, Sarah M. Glenn, Jason C. Crack, Nick E. Le Brun, José W. Saldanha, Vadim Makarov, Irene Nobeli, Kristine Arnvig, Galina V. Mukamolova, Roger S. Buxton, Jeffrey Green

**Affiliations:** 1 Molecular Biology and Biotechnology, University of Sheffield, Sheffield S10 2TN, UK; 2 School of Pharmacy, De Montfort University, Leicester LE1 9BH, UK; 3 Division of Mycobacterial Research, MRC National Institute for Medical Research, Mill Hill, London NW7 1AA, UK; 4 Department of Infection, Immunity and Inflammation, University of Leicester, University Road, Leicester LE1 9HN, UK; 5 Centre for Molecular and Structural Biochemistry, School of Chemistry, University of East Anglia, Norwich NR4 7TJ, UK; 6 Division of Mathematical Biology, MRC National Institute for Medical Research, Mill Hill, London NW7 1AA, UK; 7 A.N. Bach Institute of Biochemistry, Russian Academy of Sciences, Moscow, Russia; 8 Institute of Structural and Molecular Biology, Department of Biological Sciences, Birkbeck, University of London, Malet Street, London WC1E 7HX, UK; 9 Institute for Structural and Molecular Biology, University College London, London WC1E 6BT, UK

## Abstract

*Mycobacterium tuberculosis* (MTb) is the causative agent of pulmonary tuberculosis (TB). MTb colonizes the human lung, often entering a non-replicating state before progressing to life-threatening active infections. Transcriptional reprogramming is essential for TB pathogenesis. *In vitro*, Cmr (a member of the CRP/FNR super-family of transcription regulators) bound at a single DNA site to act as a dual regulator of *cmr* transcription and an activator of the divergent *rv1676* gene. Transcriptional profiling and DNA-binding assays suggested that Cmr directly represses *dosR* expression. The DosR regulon is thought to be involved in establishing latent tuberculosis infections in response to hypoxia and nitric oxide. Accordingly, DNA-binding by Cmr was severely impaired by nitrosation. A *cmr* mutant was better able to survive a nitrosative stress challenge but was attenuated in a mouse aerosol infection model. The complemented mutant exhibited a ∼2-fold increase in *cmr* expression, which led to increased sensitivity to nitrosative stress. This, and the inability to restore wild-type behaviour in the infection model, suggests that precise regulation of the *cmr* locus, which is associated with Region of Difference 150 in hypervirulent Beijing strains of Mtb, is important for TB pathogenesis.

## INTRODUCTION

Members of the cyclic AMP receptor (CRP)/fumarate nitrate reduction regulator (FNR) super-family of global transcription regulators control a diverse range of bacterial physiological functions. For example, different proteins in this family control aspects of carbon, nitrogen and sulfur metabolism, nitrogen fixation, aerobic and anaerobic respiration, enzymes of aromatic ring degradation and expression of virulence functions (1,2). Structural analysis has led to the definition of the archetypical CRP fold which is a versatile structure that has evolved so that members of the super-family have differing functions in signal perception, DNA-binding and interactions with RNA polymerase (3). Thus in different members of the family the signal molecules can be, for example, cyclic AMP (cAMP), oxygen, carbon monoxide, nitric oxide or 2-oxoglutarate. The best characterized member is CRP from the Gram-negative bacterium *Escherichia coli*, which controls the expression of many genes in response to changes in the cellular concentration of cAMP (4). Thus, after cAMP binding to the CRP dimer, the cAMP:CRP complex binds to promoters containing DNA sequences similar to the consensus sequence TGTGA(N_6_)TCACA (5). Subsequently CRP establishes specific protein:protein contacts with RNA polymerase that promote transcription (6). Alternatively, CRP represses transcription by promoter occlusion.

In *Mycobacterium tuberculosis*, a member of the CRP/FNR superfamily, CRP^Mt^ (encoded by gene *rv3676*), is a global transcription factor (7,8). Like the orthologous protein in *E. coli*, CRP^Mt^ binds to cAMP, but in contrast to the cAMP-dependent DNA-binding observed in *E. coli*, the cAMP-CRP^Mt^ complex exhibits only a comparatively small enhancement in DNA-binding (9). CRP^Mt^ regulates gene expression positively and negatively and is required for growth *in vitro* and in infections of macrophages and mice (8).

CRP^Mt^ is not the only member of the CRP/FNR family in *M. tuberculosis*; a second representative is coded by the *cmr* (*rv1675c*) gene (10). This protein has been found to be required for the expression of four genes previously identified as cAMP-induced genes (10,11). For three of the genes, *rv1265, groEL2* and *mdh*, the Cmr protein was shown to bind to the promoter region in electrophoretic mobility shift assays (EMSAs), subsequently confirmed in the case of *groEL2* by further EMSA and *in vitro* transcription reactions (10,12). Cmr was therefore identified as another member of the CRP/FNR family that regulates cAMP-induced genes and was accordingly given the abbreviation Cmr for cAMP and macrophage regulator, since it was required for the regulated expression of these genes during macrophage infection (10). However, Cmr binding at the *groEL2* promoter was unaffected by cAMP and transcription *in vitro* also was independent of cAMP (12).

Recently, Ranganathan *et al*. (13) reported the results of a ChIP-seq (chromatin immunoprecipitation-DNA sequencing) analysis of the Cmr homologue in *M. bovis* BCG Mb1702c (Cmr: 99% identical over 244 amino acids to *M. tuberculosis* Cmr, Rv1675c), identifying the DNA sequence recognized by Cmr and the distribution of binding sites across the *M. bovis* BCG chromosome. In addition, exposure of *M. bovis* BCG to the cAMP analogue (dibutyl-cAMP) modulated Cmr binding at a subset of chromosomal loci, including clusters of sites associated with members of the DosR regulon that exhibited co-operative binding of Cmr (13,14). The DosR regulon is required for survival during anaerobic dormancy and thus is a critical component of tuberculosis pathogenesis (14). Here, it is demonstrated that DNA-binding by Cmr is not directly modulated by cAMP but by oxidation/nitrosation of two conserved cysteine residues and that the *cmr* mutant is resistant to nitrosative stress. In addition, transcriptional profiling revealed the influence of Cmr on DosR-regulated genes in *M. tuberculosis*, suggesting a role for Cmr-mediated gene regulation in tuberculosis pathogenesis, which was supported by transient attenuation of a cmr mutant in a mouse aerosol infection model.

## MATERIALS AND METHODS

### Bacterial strains, plasmids and growth conditions

*Mycobacterium tuberculosis, Mycobacterium smegmatis* and *E. coli* strains and plasmids are listed in Supplementary Table S1. Cultures of *M. tuberculosis* (100 ml) were typically grown in 1 l polycarbonate culture bottles (Techmate) in a Bellco roll-in incubator (two revolutions per minute (rpm)) at 37°C in Dubos broth containing 0.05% (v/v) Tween 80 supplemented with 0.2% (v/v) glycerol and 4% Dubos medium albumin. Aerosol infections of mice were carried out as described previously (15). Cultures of *E. coli* and *M. smegmatis* mc^2^ 155 (500 ml) were typically grown in Lennox broth medium (16) in a 1:4 volume/flask ratio at 37°C with shaking at 250 rpm.

### Generation of an unmarked *cmr M. tuberculosis* deletion mutant

Approximately 1 kb upstream and downstream of *cmr* were amplified by PCR using oligonucleotides 1675c_fwd1 (5΄-CCGGTCGACCGCTGAGACTGGTTTATG-3΄) and 1675c_rev1 (5΄-CAATGACACAGCGCCGGTCCCAC-3΄), and 1675c_fwd2 (5΄-TGCCATGGCCCCTCCTTGAGAGC-3΄) and 1675c_rev2 (5΄-CGGTCGACAAGTCGTGCCCATCAAG-3΄) (restriction enzyme sites used for cloning are underlined), and fused in a consecutive PCR using oligonucleotides 1675c_fwd1 and 1675c_rev2. The resulting 2 kb product was cloned into vector p2NIL creating plasmid p2NIL:1675c and verified by sequencing. A PacI fragment from the vector pGOAL17 containing P_Ag85_-*lacZ*, P_hsp60_-*sacB* was ligated into the single PacI site in p2NIL:1675c creating plasmid p2NIL:1675c.17.

Competent *M. tuberculosis* H37Rv cells were electroporated with 1 μg of plasmid p2NIL:1675c.17 and transformants were selected on 7H11 medium plus kanamycin (25 mg l^−1^) and 5-bromo-4-chloro-3-indolyl-β-d-galactopyranoside (X-Gal; 40 mg l^−1^). Blue colonies (single cross-overs) were re-streaked on 7H11-plates without antibiotics to allow for recombination. A loopful of cells was suspended and serial dilutions plated onto 7H11 + X-Gal + sucrose (2%). Suc^R^ (sucrose resistant) colonies were then streaked onto plates with and without kanamycin to identify Kan^S^ (kanamycin sensitive) colonies. DNA from four potential *cmr* mutants was isolated using Instagene matrix (BioRad) and subjected to PCR analysis. One of the potential double crossovers was identified as a mutant of *cmr* by PCR, and verified by DNA sequencing over the *cmr* region. Genomes of the *cmr* mutant and wild type *M. tuberculosis* were sequenced by GATC Biotech using an Illumina MiSEq platform.

### Construction of a *cmr* complementing plasmid

A *cmr* complementing construct with expression of the gene under its own promoter was prepared by PCR of genomic DNA using the following primers: 5΄-GCGGTACCCTCGCGGTACTGCACTCGGT-3΄ and 5΄-GCGAATTCTCATTGAGCCCGGGCGCG-3΄ (restriction enzyme sites underlined). The region amplified contained the *cmr* gene and the region 500 bp upstream. After digestion with KpnI and EcoRI this product was cloned into the plasmid pKP186. This plasmid was verified by DNA sequencing (GATC Biotech) and confirmed to correspond to the base pairs 1900241–1901475 on the *M. tuberculosis* H37Rv genome. The confirmed plasmid was integrated into the chromosome using pBS-int as described previously (8).

### cDNA labelling and transcriptomic analysis

Cultures of *M. tuberculosis* H37Rv, the *cmr* deletion mutant and a complemented mutant were grown aerobically in Dubos medium supplemented with 0.05% Tween 80, 0.2% glycerol and 4% Dubos medium albumin to mid-exponential phase (OD_600_ ∼0.6). RNA isolation from *M. tuberculosis* liquid cultures was described previously (17). Whole genome DNA microarrays of *M. tuberculosis* (version 2) were provided by the BμG@S group (St. George's, University of London). cDNA labelling and RNA-DNA microarray hybridizations were described previously (8). Microarray slides were scanned as previously discussed (18) and image quantitation performed using Bluefuse for Microarrays v3.6 (BlueGnome). Three biological replicates were performed for each condition, carried out in duplicate for dye-swaps. Thus, data were obtained from six slides, including dye swaps, from three bacterial cultures. Data were analysed using GeneSpring version 13 (Agilent), applying a global Lowess normalization to remove differences in dye-incorporation efficiencies between microarrays. Features with a Bluefuse confidence of <0.1 (a quality control metric utilized by the software to quantify spot quality) were eliminated from further analysis. Genes were considered to be altered if they showed >3-fold change in absolute expression and passed significance filtering by using a *t*-test (*P*-value <0.1) applying a Benjamini and Hochberg multiple testing correction. The array design is available in ArrayExpress (accession number A-BUGS-23). Fully annotated microarray data have been deposited in ArrayExpress (accession no. E-BUGS-155).

### Overproduction and purification of Cmr

Cmr protein was overproduced with an N-terminal hexa-His-tag in *E. coli* (BL21-λDE3) from a pET28a derivative pGS2103 (Supplementary Table S1). Cultures were grown at 37°C to an optical density (OD) of ∼0.6, when IPTG (isopropyl β-d-thiogalactoside; 120 μg ml^−1^) was added and the cultures incubated at 25°C for a further 2 h. Cells were lysed after resuspension in 20 mM sodium phosphate buffer (pH 7.4) containing 0.5 M NaCl by two passages through a French pressure cell at 37 MPa. The cell-free extract (resulting from centrifugation of the lysate) was applied to a 1 ml Hi-Trap Chelating column (GE Healthcare) and the recombinant Cmr isolated by elution with a linear imidazole gradient (12).

### Partial proteolysis

Trypsin digests of Cmr were optimized and the following conditions used. Trypsin (Sigma) was used at a final concentration of 2.1 μM in 10 mM Tris–HCl (pH 8.3), with 3.3 μM Cmr for 1 min at room temperature with and without a 10-fold molar excess of cAMP (or other potential signal molecules). Reactions were stopped by the addition of 10% SDS and heated for 10 min at 100°C before analysis by sodium dodecylsulfate polyacrylamide gel electrophoresis (SDS-PAGE).

### Electrophoretic mobility shift assays (EMSAs) and *in vitro* transcription

EMSAs were performed using the *cmr* promoter (P*cmr*) region (unless stated otherwise). P*cmr* was released from pGS2462 (Supplementary Table S1) using EcoRI as a fragment extending from –174 bp to +56 bp relative to the start codon. The DNA fragment was end labelled using 0.37 MBq of [α-^32^P]dCTP and Klenow enzyme (10 units) for 60 min at room temperature. Unincorporated radionucleotides were removed using a QIAquick PCR clean-up kit (Qiagen). Increasing concentrations of recombinant Cmr in the presence of 100 mM NaCl, 40 mM Tris–HCl (pH 8), 10 mM MgCl_2_, 1 mM ethylenediaminetetraacetic acid (EDTA), 250 μg ml^−1^ bovine serum albumin (BSA) and 1 μg calf thymus DNA (300-fold excess) (12) were incubated with different stress reagents as indicated (either DTT, 5 mM; diamide, 5 mM; or *S*-nitrosoglutathione (GSNO), 1 mM) for 30 min at room temperature. Radiolabelled DNA (∼3 ng) was then added to the reactions and incubated for 10 min at room temperature before the resulting complexes were separated on 6% (w/v) polyacrylamide gels buffered with Tris-glycine. Similar protocols were followed for EMSAs with DNA fragments upstream of the *groEL2, rv2007c, rv2032, rv3133c* and *rv3134c* genes released from pCR4BLUNT-TOPO derivatives (Supplementary Table S1; see *Supplementary information* for all DNA sequences used in EMSAs).

*Mycobacterium smegmatis* RNA polymerase (RNAP) holoenzyme was isolated from *M. smegmatis* mc^2^ 155 as described (19). The *rrnA* and *cmr* promoter DNA fragments were amplified from *M. tuberculosis* H37Rv genomic DNA by PCR using primers *rrnA_F* (5΄-TAGAGCAATTCGAACGGGTATGCTG-3΄) and *rrn_R* (5΄-TAGAGAATTGCTGTGAAACCACCAAACA-3΄) and primers *cmr_F* (5΄-TAGAGAATTCGTCCACCGGTTG-3΄) and *cmr_R* (5΄-TAGAGAATTCGAGCAGGCGATG-3΄) respectively. Transcription was performed in 10 μl transcription buffer (TB; 20 mM Tris–HCl, pH 8.0, 20 mM KCl, 10 mM MgCl_2_ and 5% (v/v) glycerol). Typically a promoter fragment (20 nM) was incubated with Cmr at the indicated concentrations for 10 min at 37°C. RNAP holoenzyme (200 nM) was added and the mixture was incubated for 10 min at 37°C. Transcription was initiated by the addition of 250 μM corresponding initiating NTPs, 10 μM non-initiating NTPs and radiolabelled [α-^32^P] NTP as indicated. The reactions were stopped by adding 1 volume of stop buffer (1× TBE, 8 M urea). Products were resolved by denaturing PAGE (8 M urea), visualized by PhosphorImaging (GE Healthcare) and analyzed using ImageQuant software (GE Healthcare). The DNA sequences of the templates used in the *in vitro* transcription reactions are provided in the *Supplementary information*.

### Bio-layer interferometry (BLItz)

P*cmr* was amplified from pGS2462 using a primer with a 5΄-biotin label (Supplementary Table S1), and the resulting DNA was extracted from a 1% (w/v) agarose gel using the QIAquick gel extraction kit (Qiagen). The extracted DNA was typically used at a concentration of 56 ng μl^−1^ for binding to the streptavidin probe. Cmr (452 nM) was typically treated with a stress agent (as indicated) for ∼2 h before binding to the probe. The association and dissociation rate constants at 20°C were calculated by curve fitting using the BLItz software (ForteBio).

### Mass spectrometry

Cmr samples (∼1 mg ml^−1^; ∼40 μM) for liquid chromatography–mass spectrometry (LC–MS) were treated with appropriate stress agents (∼20 mM dithiothreitol (DTT) or diamide) for 45 min on ice. Samples were then diluted to (0.1 mg ml^−1^; ∼4 μM) with an aqueous mixture of 1% (v/v) acetonitrile, 0.3% (v/v) formic acid, and loaded onto a ProSwift RP-1S column (4.6 × 50 mm) (Thermo Scientific) on an Ultimate 2000 uHPLC system (Dionex, Leeds, UK). Bound proteins were eluted (0.2 ml min^−1^) using a linear gradient (15 min) from 1% to 100% (v/v) acetonitrile, 0.1% (v/v) formic acid. The eluent was continuously infused into a Bruker microQTOF-QIII mass spectrometer, running Hystar (Bruker Daltonics, Coventry, UK)), using positive mode electrospray ionization (ESI). Compass Data Analysis, with Maximum Entropy v1.3, (Bruker Daltonics, Coventry) was used for processing of spectra under the LC peak. The mass spectrometer was calibrated with ESI-L tuning mix (Agilent Technologies). Alternatively, for LC-MS of Cmr before and after treatment with 20-fold molar excess of GSNO for 1 h, samples were diluted with Solvent A (water with 0.1% (v/v) formic acid) and loaded onto an Agilent Zorbax Extended-C18 (2.1 × 50 mm) column. Bound proteins were eluted (0.4 ml min^−1^) using a gradient (10 min) of solvent B from 5% to 95% (v/v) acetonitrile, 0.1% (v/v) formic acid. In this case, analysis was carried out using an Agilent Technologies 6530 Q-ToF LC–MS instrument, at The University of Sheffield Faculty of Science Mass Spectrometry Centre.

### *Mycobacterium tuberculosis* stress responses

*Mycobacterium tuberculosis* strains were grown in 7H9 Middlebrook broth supplemented with 10% (v/v) albumin-dextrose complex (ADC), 0.2% (v/v) glycerol and 0.05% (w/v) Tween 80 at 37°C with shaking (100 rpm) until mid-exponential growth phase corresponding to OD_580nm_ ∼0.5–0.6. The nitric oxide (NO) donor, 6-methoxy-5-nitropyrimidin-4-yl pyrrolidine-1-carbodithioate (20), at a final concentration of 100 μM was used to assess the effect of nitrosative stress on *M. tuberculosis* viability. For starvation of *M. tuberculosis*, cultures were washed in phosphate buffered saline (PBS) twice before being resuspended in PBS. These cultures were left static at 37°C for up to 6 weeks. Mycobacterial viability following each of these stresses was assessed by measuring the number of colony forming units (cfus) after static incubation at 37°C. Percentage survival was calculated as the ratio of cfu after treatment to cfu before treatment multiplied by 100. Control chemicals which do not release nitric oxide had no effect on mycobacterial viability. The minimum inhibitory concentrations (MICs) of the NO donor were also assessed, using the micro-dilution method. 7H9 Middlebrook broth supplemented with 10% (v/v) albumin-dextrose complex, 0.2% (v/v) glycerol and 0.05% (w/v) Tween 80 containing 0.005% (w/v) of resazurin supplemented with the NO donor (0-4 μg ml^−1^ final concentration) was inoculated with *M. tuberculosis* bacilli (∼5 × 10^4^). Growth of bacteria was monitored visually by the change of blue colour to pink and turbidity. The first concentration that completely inhibited mycobacterial growth was considered as the MIC.

### Growth of *M. tuberculosis* in murine bone marrow-derived macrophages

Growth of macrophages was carried out as described previously (15). *Mycobacterium tuberculosis* H37Rv, *cmr* mutant and the complemented *cmr* mutant were grown in 7H9+ADC medium (see above) to OD_600_ ∼0.4–0.8 in roller bottles, centrifuged at 3000 rpm for 5 min, washed twice with PBS + 0.05% (v/v) Tween 80 and centrifuged at 1200 rpm for 5 min to remove clumps. The concentration was adjusted as required assuming OD_600_ of 1.0 is 5 × 10^7^ cfu ml^−1^, and dilutions were plated on agar to confirm exact cfu. Macrophages (2 × 10^5^) were infected at a moi (multiplicity of infection) of 1.0 for 4 h. Experiments were repeated three times.

### Growth of *M. tuberculosis* in mice

*Mycobacterium tuberculosis* cultures were grown in 7H9 medium to an OD_600_ of ∼0.6. Cultures were pelleted and resuspended in Dulbecco's modified Eagle's medium (DMEM, Sigma) plus 2 mM l-glutamine and 50% (v/v) fetal calf serum to a concentration of 10^9^ bacteria per ml. Subsequently, 10 ml bacterial stocks were prepared containing ∼10^5^ cfu ml^−1^, and mice were infected with ∼100 cfu each, using a Glas-Col aerosol infection system. Aerosol infections were performed at the National Institute for Medical Research (Mill Hill, UK). The infection was monitored by removing the lungs and spleens of infected mice at various intervals, homogenizing the tissues, and plating 10-fold serial dilutions on 7H11–OADC agar plates (Becton, Dickinson and Company) to determine the cfu values. The results for each time point are the means of cfu determinations performed on organs from five mice, and the error bars show the standard error of the mean. Experiments involving mice were conducted in strict accordance with the UK Animal (Scientific Procedures) Act 1986 under project license 80/2236 and all efforts were made to minimize suffering. Female, specific pathogen free Balb/c mice (6–8 weeks old) were obtained from the breeding facility at the National Institute for Medical Research and the studies were performed in containment category 3 animal facilities.

## RESULTS AND DISCUSSION

### DNA-binding and partial proteolysis of Cmr is unaffected by cAMP

**Figure 1.**
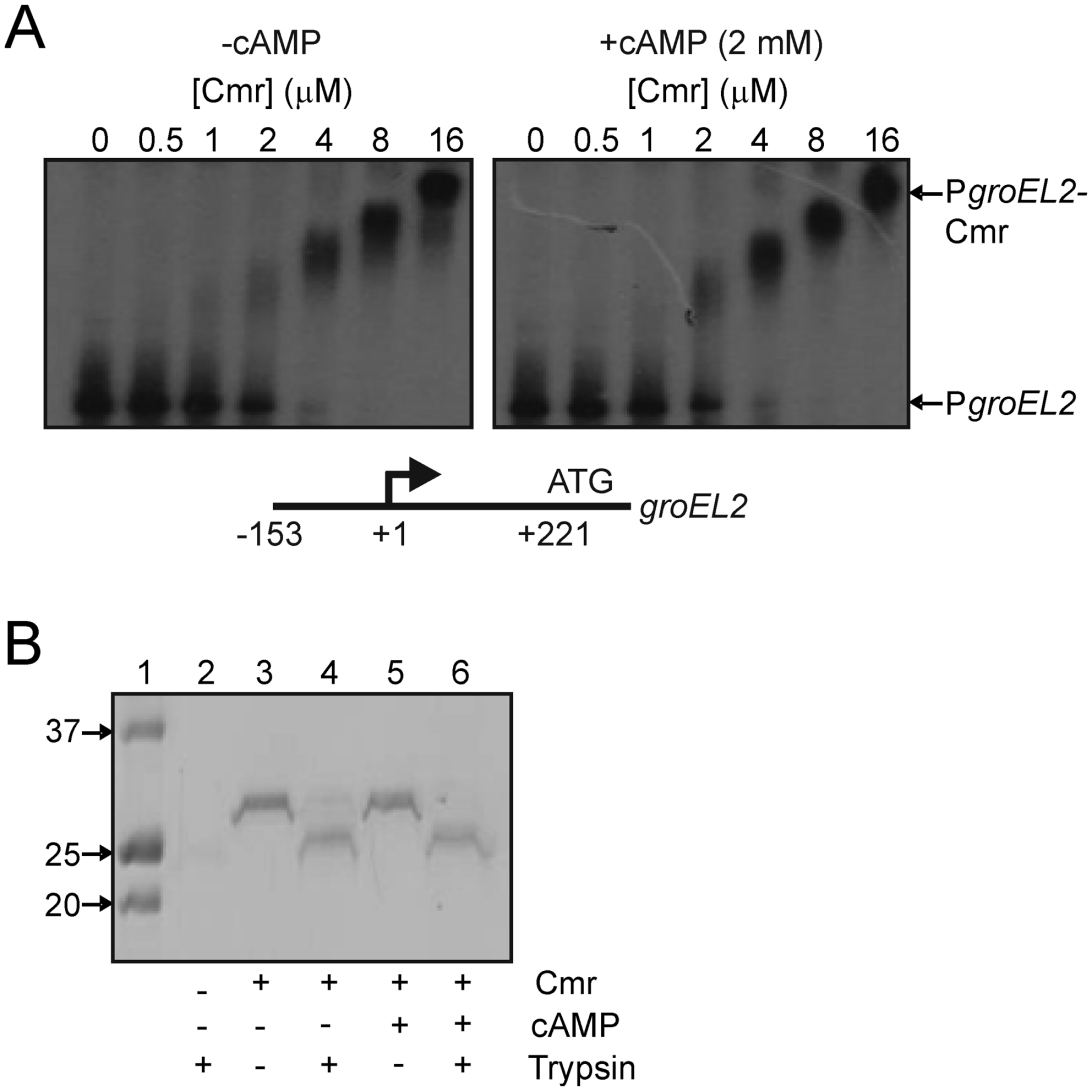
Cmr binding at the *groEL2* promoter (P*groEL2*) is independent of cAMP and Cmr does not bind cAMP. (**A**) Radiolabelled P*groEL2* (amplified from p2126, Supplementary Table S1) was incubated with increasing concentrations of Cmr, with or without cAMP (2 mM final concentration) as indicated, before separation of protein-DNA complexes (P*groEL2*-Cmr) from free DNA (P*groEL2*) by electrophoresis. The final concentration of Cmr dimer in the assays is indicated above each lane. A diagram (not to scale) showing features (arrow, transcript start; ATG, *groEL2* translational start) of the DNA used in the EMSAs is shown below the autoradiographs. (**B**) Partial proteolysis of Cmr in the presence and absence of a 10-fold molar excess of cAMP. Lane 1, Precision Plus Protein Standards (BioRad) with molecular weights in kDa indicated; Lanes 2–6, reaction components are as indicated below the image of the gel.

*Mycobacterium tuberculosis* H37Rv possesses two members of the CRP family of transcription factors, Cmr (Rv1675c) and Rv3676 (21). Gel filtration analysis, and chemical cross-linking both indicated that, like most other members of the CRP/FNR superfamily, Cmr is dimeric (Supplementary Figure S1). *Escherichia coli* CRP and *M. tuberculosis* Rv3676 are global regulators that bind cAMP (22). However, Cmr lacks 15 of the 17 amino acids that form the primary cAMP binding pocket of Rv3676 (23). The secondary cAMP-binding site in Rv3676 (composed of Asn67, Asn137, Asp140 and Gln156) is also poorly conserved in Cmr. Thus, although associated with regulation of cAMP- and macrophage-induced genes, it was concluded that Cmr was unlikely to interact with cAMP. Consistent with this view Cmr binding to several potential target promoters was unaffected by addition of cAMP (10,12). Here, Cmr binding at the *groEL2* promoter region was unaffected by addition of 2 mM cAMP and partial proteolysis of Cmr in the presence and absence of cAMP (and other related nucleotides: AMP, ADP, ATP, cCMP, CMP, CDP, CTP, UMP, UDP and UTP) yielded identical profiles, indicating that Cmr does not respond to cAMP or any of the other nucleotide ligands tested (Figure 1). Thus, although *M. bovis* BCG Cmr has been associated with cAMP-responsive gene regulation *in vivo* (10), there is no evidence in favour of direct interaction between cAMP and *M. tuberculosis* Cmr (this work) or *M. bovis* BCG Cmr (13, unpublished data). Other members of the CRP family have been shown to utilize heme and iron-sulfur clusters as sensory co-factors (2), but the UV-visible spectrum of isolated Cmr did not indicate the presence of these co-factors, nor did Cmr specifically incorporate heme or iron in reconstitution reactions. Thus, it was concluded that Cmr does not bind cAMP and lacks cofactors that could function as sensory modules.

### Transcriptional profiling of *cmr* mutant

Comparison of the transcript profiles of aerobic mid-exponential (OD_600_ ∼0.6) laboratory cultures of *M. tuberculosis* H37Rv and the *cmr* mutant showed that several members of the DosR regulon were up-regulated compared to the parental strain (Table 1). Thus, all the >3-fold up-regulated genes in the *cmr* mutant were members of the Dos regulon and the top the eight transcripts with the highest induction ratios in the *cmr* mutant corresponded to those most up-regulated under hypoxia, albeit in a different order (Supplementary Table S2; 24). Transcript profiling of the *M. tuberculosis cmr* mutant compared with the same strain transformed with an integrated plasmid expressing *cmr* resulted in extensive complementation of the mutant transcription profile, indicating that the observed changes in transcription were mediated by disruption of the *cmr* gene (Table 1). Previous transcript profiling data obtained by 6-fold over-expression of *cmr* suggested that no genes were significantly regulated (≥2-fold, *P* ≤ 0.01) (25), hence, the *cmr* mutant to parent comparison undertaken here has revealed components of the Cmr regulon under laboratory conditions for the first time. Although the changes in transcript abundance reported by Rustad *et al*. did not meet the statistical criteria to be considered significant, nine Cmr-repressed genes (*rv0080, rv1738, rv2007c, rv2031c, rv2032, rv2623, rv2626c, rv3130c, rv3131*) detected here were also repressed by Cmr when *cmr* was over-expressed (25).

**Table 1.** Microarray data for the *M. tuberculosis* genes whose expression was altered >3-fold upon *rv1675c* deletion, compared to the wild-type

Rv number	Gene name	Gene product	DosR regulon	*cmr* mutant/parent		*cmr* mutant/complemented *cmr* mutant		Complemented *cmr* mutant/parent	
				log_2_ ratio	*P* value	log_2_ ratio	*P* value	log_2_ ratio	*P* value
Rv0079	*rv0079*	Unknown protein	Yes	**2.08**	**0.035**	**2.53**	**0.033**	0.45	0.623
Rv0080	*rv0080*	Conserved hypothetical protein	Yes	**2.07**	**0.005**	2.51	N/A	0.44	N/A
Rv1675c	*cmr*	Probable transcriptional regulatory protein Cmr		**–2.87**	**0.001**	–1.85	0.203	1.02	0.013
Rv1737c	*narK2*	Possible nitrate/nitrite transported NarK2	Yes	**1.58**	**0.038**	**2.01**	**0.077**	0.42	0.582
Rv1738	*rv1738*	Conserved protein	Yes	**2.24**	**0.032**	**2.35**	**0.003**	0.11	0.921
Rv2007c	*fdxA*	Ferredoxin FdxA	Yes	**3.09**	**0.023**	**2.72**	**0.000**	–0.37	0.773
Rv2030c	*rv2030c*	Conserved protein	Yes	**3.05**	**0.015**	**2.88**	**0.009**	–0.16	0.865
Rv2031c	*hspX*	Stress protein induced by hypoxia	Yes	**3**	**0.035**	**3.91**	**0.000**	0.92	0.522
Rv2032	*acg*	Conserved protein Acg	Yes	**1.88**	**0.031**	**2.29**	**0.019**	0.41	0.657
Rv2623	*TB31.7*	Universal stress protein	Yes	**1.67**	**0.035**	1.28	0.222	–0.39	0.564
Rv2626c	*hrp1*	Hypoxic response protein 1 Hrp1	Yes	**2.4**	**0.093**	**2.70**	**0.002**	0.30	0.809
Rv2629	*rv2629*	conserved hypothetical protein	Yes	**2**	**0.018**	1.00	0.227	–0.99	0.170
Rv3127	*rv3127*	conserved hypothetical protein	Yes	**1.64**	**0.074**	**2.04**	**0.025**	0.39	0.696
Rv3130c	*tgs1*	Triacylglycerol synthase (diacylglycerol acyltransferase) Tgs1	Yes	**2.55**	**0.030**	**4.42**	**0.099**	**1.87**	**0.078**
Rv3131	*rv3131*	Conserved protein	Yes	**2.63**	**0.040**	**3.00**	**0.002**	0.37	0.764
Rv3133c	*dosR*	Two component transcriptional regulatory protein DosR	Yes	**1.93**	**0.031**	**1.77**	**0.023**	–0.16	0.872
Rv3134c	*rv3134c*	Universal stress protein family protein	Yes	**1.61**	**0.032**	1.75	0.105	0.13	0.857

Values in bold and underlined indicate significance using a *t*-test (*P* value ≤ 0.1). N/A indicates insufficient data for significance testing.

### Cmr is an auto-regulator and binds at the *dosR* promoter region

**Figure 2.**
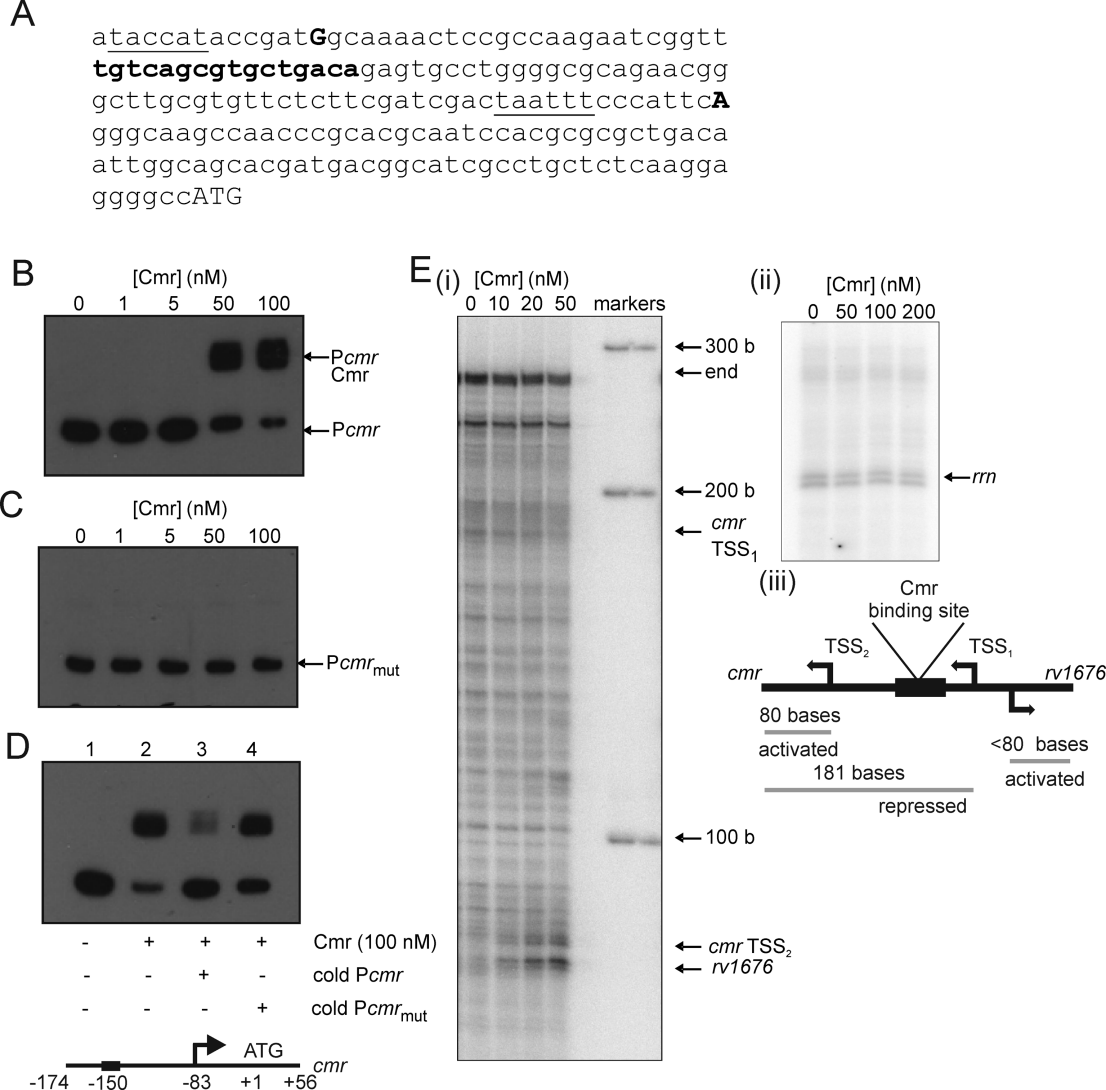
Cmr binds to its own promoter in a site-specific manner to repress transcription. (**A**) DNA sequence covering the *cmr-rv1676* intergenic region. The locations of TSSs (bold upper case), potential –10 elements (underlined), the Cmr-binding site (bold lower case) and the *cmr* start codon (ATG) are indicated. (**B**) Radiolabelled P*cmr* was incubated with increasing concentrations of Cmr before separation of protein-DNA complexes (P*cmr*-Cmr) from free DNA (P*cmr*) by electrophoresis. (**C**) Site-directed mutagenesis of the predicted Cmr binding site (GTCAGCGTGCTGCA to ATTAGCGTGCTAAT; P*cmr*_mut_) in P*cmr* abolishes Cmr binding. For (B) and (C), the final concentrations of Cmr in the assays are indicated. (**D**) Competition assays in which binding of Cmr (100 nM) to radiolabelled P*cmr* (lane 2) was challenged by the presence of a 5-fold molar excess of unlabelled P*cmr* (lane 3) or 5-fold molar excess of unlabelled P*cmr*_mut_ (lane 4). A diagram (not to scale) showing features (arrows, transcript starts; ATG, cmr translational start) of the DNA locus used in the EMSAs is shown below panel D. (**E**) *In vitro* transcription from the *cmr-rv1676* intergenic region. (i) A 282 bp DNA fragment containing the *cmr-rv1676* intergenic region was incubated with *M. smegmatis* RNA polymerase along with the indicated concentrations of Cmr protein. Transcription reactions were carried out as described in the *Materials and Methods*. The locations of the *cmr* transcripts (TSS_1_, TSS_2_; Figure 2A), the *rv1676* transcript (*rv1676*) and the sizes of the calibrating RNA ladder (bases, b) are indicated. (ii) Control reactions were carried out using the *rrnA* promoter in the presence of the indicated concentrations of Cmr. (iii) A schematic diagram of transcription regulation at the *cmr-rv1676* intergenic region. Transcript starts (TSS, arrows), transcripts (grey lines), transcript sizes and whether transcription is activated or repressed are indicated.

RNA-seq identified two weak transcript start sites (TSSs), associated with plausible –10 elements, in the *cmr* gene (26). The *cmr* TSSs are located 183 (TSS_1_) and 82 (TSS_2_) bp upstream of the *cmr* start codon (Supplementary Figure S2A). An inverted repeat (TGTCAGCGTGCTGACA) related to the Cmr consensus proposed by Ranganathan *et al*. (13) was identified between the two *cmr* TSSs (Figure 2A). Cmr binding at this location was shown by EMSAs (Figure 2B). Mutation of four bases in the palindromic regions of the predicted binding site (TGTCAGCGTGCTGACA to T**A**T**T**AGCGTGCT**A**A**T**A altered bases in bold) abolished DNA-binding by Cmr (Figure 2C). This sequence-specific binding was confirmed by competition experiments in which a 5-fold excess of unlabelled *cmr* promoter DNA (P*cmr*) inhibited binding of Cmr to radiolabelled P*cmr* (Figure 2D, compare lanes 2 and 3), whereas 5-fold unlabelled P*cmr* containing the mutated binding site did not (Figure 2D, compare lanes 2 and 4). *In vitro* transcription assays confirmed that transcription of *cmr* is weak; end run-off transcripts resulting from non-specific transcription initiation from the ends of the linear 282 bp DNA fragment containing the *cmr-rv1676* intergenic region were relatively strong and unaffected by the presence or absence of Cmr. However, one specific transcript consistent with a size of 181 bases was produced in the presence of RNA polymerase, and assigned as the *cmr* transcript originating from TSS_1_ (Figure 2A and E). Addition of Cmr protein resulted in dose-dependent inhibition of transcription from TSS_1_ while the levels of two shorter transcripts increased; the first (consistent with a size of 80 bases) was assigned as originating from TSS_2_ and the second (consistent with a size of ≤80 bases) represented the divergent *rv1676* transcript (Figure 2E, i; Supplementary Figure S2B). This suggests that Cmr can recruit RNA polymerase to activate transcription from divergent promoters as previously observed for the *E. coli* CRP protein (27). These regulatory events were judged to be specific because Cmr had relatively little effect on transcription from the *rrnA* promoter 3 (Figure 2E, ii). Additional evidence showing that Cmr acts independently of cAMP was provided by Cmr-dependent transcription activation being unaltered in the presence or absence of cAMP (Supplementary Figure S2C). Thus, like Cmr-mediated repression (12), Cmr-dependent transcription activation was cAMP-independent. Thus, it was concluded that Cmr binds at the *cmr-rv1676* intergenic region to repress the upstream *cmr* promoter and activate both the downstream *cmr* promoter and the divergent *rv1676* promoter independently of cAMP (Figure 2E, iii).

**Figure 3.**
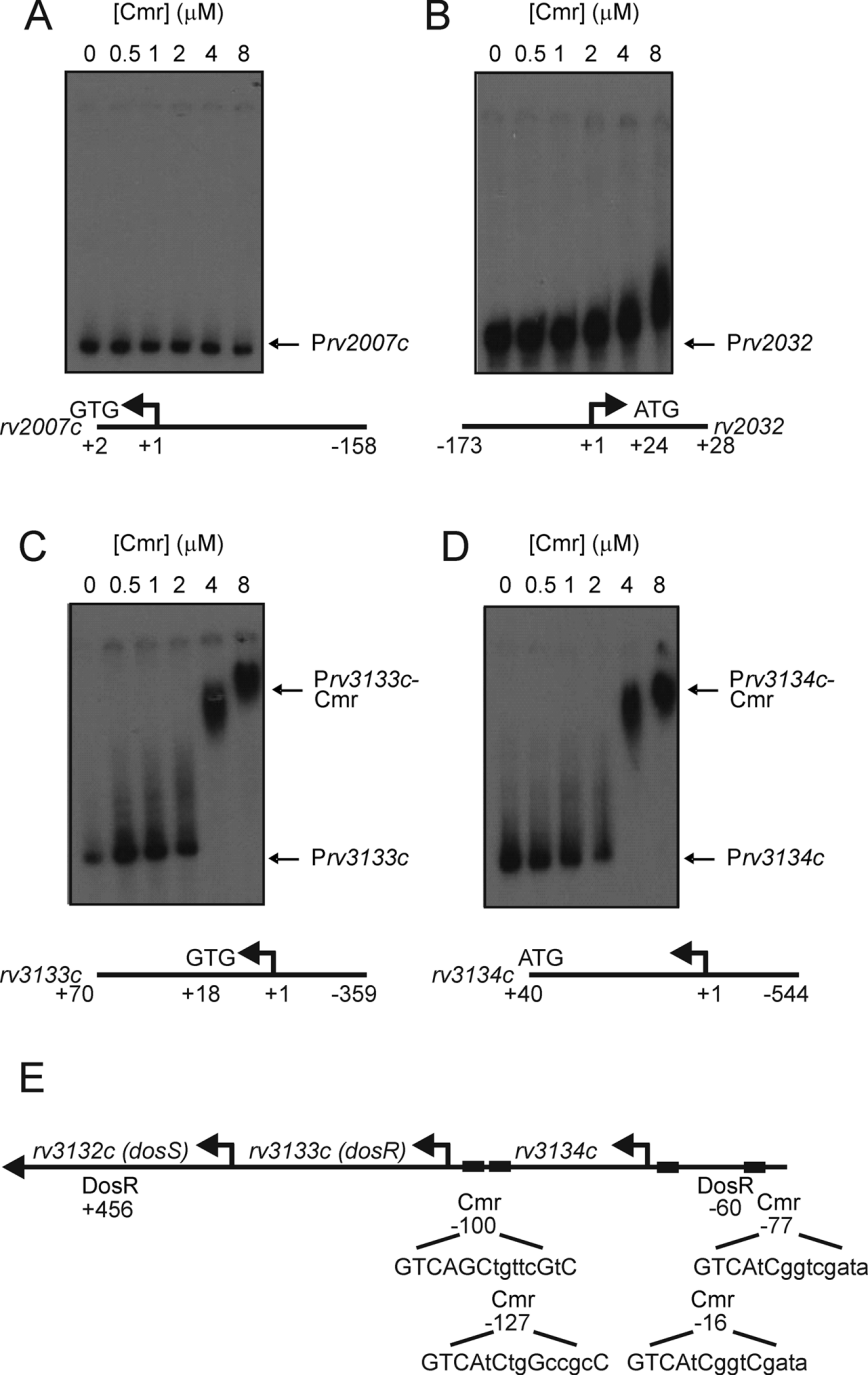
Cmr binds at the *rv3134c-dosR-dosS* region but not at all DosR-regulated promoters. Radiolabelled DNA fragments amplified from the regions upstream of (**A**) *rv2007c*, (**B**) *rv2032*, (**C**) *rv3133c* and (**D**) *rv314c* were incubated with the indicated concentrations of Cmr before separation of protein–DNA complexes from free DNA by electrophoresis. The locations of free DNA and Cmr complexes are indicated. Below each image is a schematic representation (not to scale) of the DNA fragments used in the EMSA (DNA sequences are provided in *Supplementary information*). Numbering is relative to the locations of the transcript starts (+1, arrows; 26). Start codons are also shown. (**E**) Schematic representation (not to scale) of the *rv3134c-dosR-dosS* locus. The locations (relative to the adjacent transcript starts) of DosR and predicted Cmr binding sites (boxes) are shown. Bases that match the Cmr consensus in the predicted Cmr sites are indicated in upper case.

The DosS/T-DosR system responds to changes in redox state, responding to hypoxia, nitric oxide and carbon monoxide (28). Transcript profiling suggested that several members of the Dos regulon (including *dosR, rv3133c*) were regulated by Cmr (Table 1). It was reasoned that if Cmr was a repressor of *dosR* transcription, mutation of *cmr* would relieve this repression and lead to the observed up-regulation of DosR regulon genes. Therefore, four genes were selected from amongst those exhibiting differential expression patterns in the *cmr* mutant (Table 1) (*rv2007c, rv2032, rv3133c* and *rv3134c*), and the DNA sequences upstream of the coding regions were used to test the hypothesis that Cmr exerts control over the DosR regulon by regulation of *dosR* expression. Whilst Cmr was unable to bind upstream of *rv2007c* or *rv2032*, interaction was observed with DNA upstream of *dosR* (*rv3133c*) and *rv3134c* (Figure 3). The latter is co-transcribed as an *rv3134c-dosR-dosS* transcript (29). Analysis of the DNA sequence upstream of *rv3133c* and*rv3134c* revealed that both contained tandem sequences similar to the Cmr binding site in P*cmr* (cGTCAtCtggccGcCA and gGTCAgCtgttcGtCg for *rv3133c*; and TGTCAtcggtcgataA and gGTCcccgccagtAac for *rv3134c*; consensus bases in upper case), whereas *rv2007c* and *rv2032* lacked such sequences (Figure 3E; Supplementary Figure S3). The locations of the putative Cmr sites (–77, –16, +740 and +767 relative to the *rv3134c* transcript start) are consistent with the proposal that Cmr represses expression of the *rv3134c-dosR-dosS* operon.

### Cmr is a redox sensor

**Figure 4.**
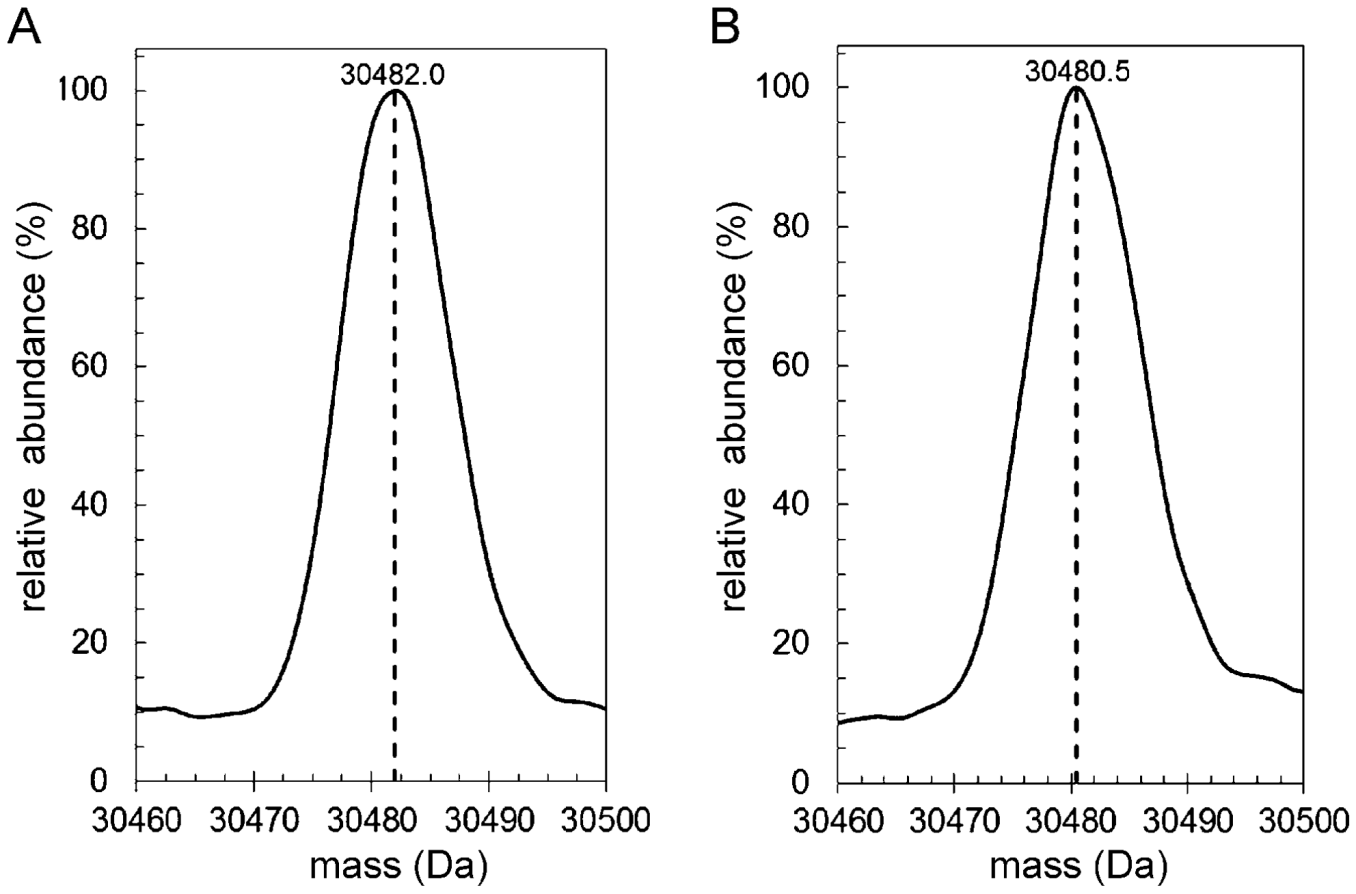
Liquid Chromatography Mass Spectrometry analysis of Cmr. Cmr analyzed in the (**A**) reduced (DTT-treated) and (**B**) oxidized (diamide-treated) states.

The dysregulation of the DosR regulon in the *cmr* mutant implicated Cmr in redox sensing. Further evidence linking the Cmr to redox homeostasis is provided by the location of the *cmr* gene itself. It is divergently transcribed from *rv1676* and *dsbF* (*rv1677*) and Cmr activates expression of *rv1676* in vitro (Figure 2E). DsbF is a thiol oxidase that is required for the correct folding of surface- and secreted-proteins that contain disulfide bonds (30). Rv1676 has a peroxiredoxin-like 2 domain with potential role in anti-oxidant defence under aerobic conditions (30). Accordingly, *rv1676* transcripts were detected in exponential aerobic cultures of *M. tuberculosis* (Supplementary Figure S2A). Cmr has two cysteine residues (Cys36 and Cys131). Liquid chromatography–mass spectrometry (LC–MS) showed that reduced Cmr had the mass (30482.0 Da, including the His-tag) predicted from the amino acid sequence (Figure 4A). Treatment with diamide, which promotes disulfide bond formation in susceptible proteins, gave a mass of 30480.5 Da, suggesting a loss of two protons, consistent with formation of an intramolecular disulfide bond between Cys36 and Cys131 (the only Cys residues in Cmr), suggesting the possibility that Cmr acts as a redox sensor via a dithiol-disulfide switch (Figure 4B).

**Figure 5.**
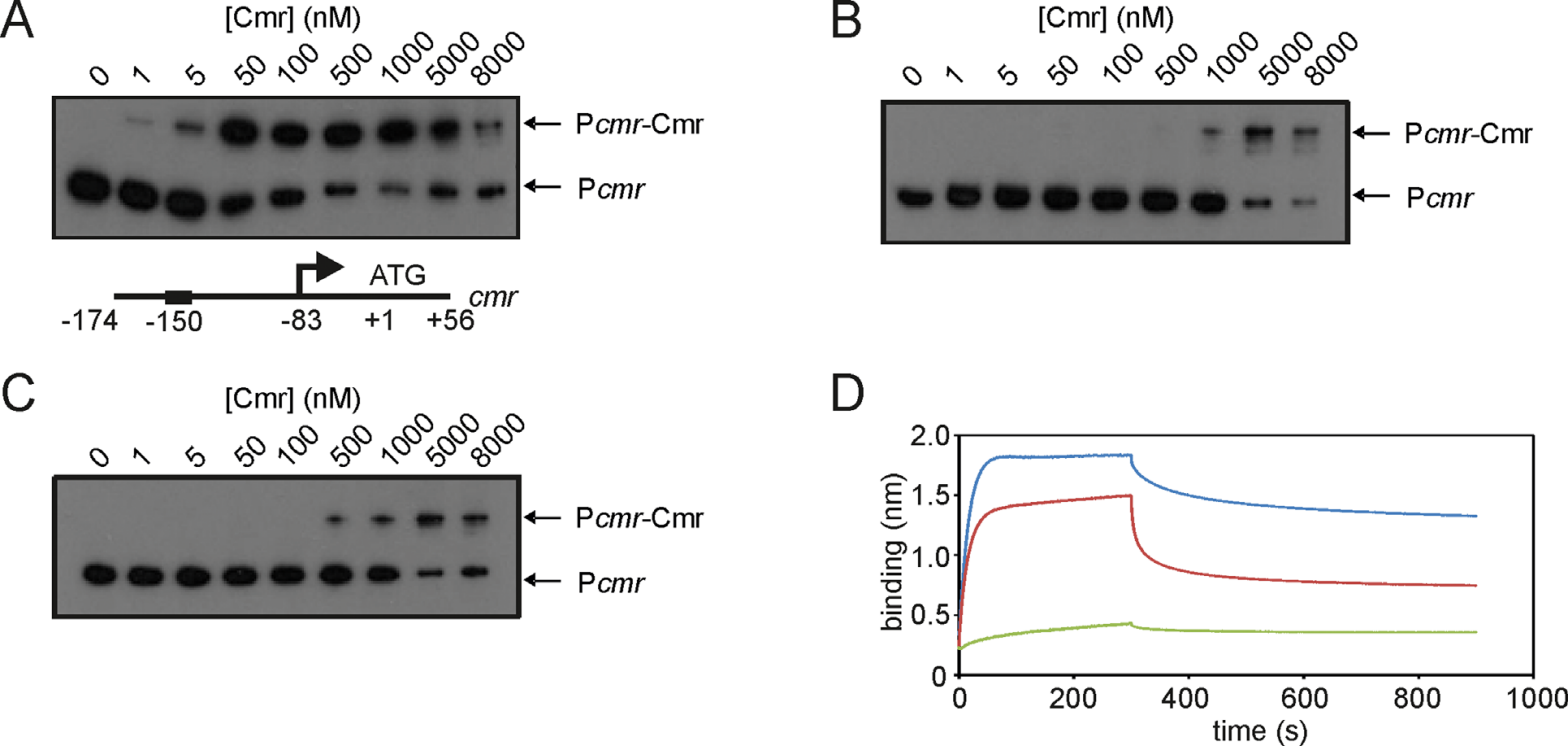
DNA-binding by Cmr is affected by its redox state. Radiolabelled P*cmr* was incubated with the indicated concentrations of Cmr before separation of protein–DNA complexes (P*cmr*-Cmr) from free DNA (P*cmr*) by electrophoresis. Reactions contained: (**A**) 5 mM DTT; (**B**) 5 mM diamide; (**C**) 5 mM hydrogen peroxide (H_2_O_2_). A diagram (not to scale) showing features (arrows, transcript starts; ATG, *cmr* translational start) of the DNA locus used in the EMSAs is shown below panel A. (**D**) BLItz assays. Biotin-labelled P*cmr* bound to a streptavidin probe was exposed to reduced (blue line), oxidized (red line) or nitrosated (green line) Cmr protein (452 nM).

The effect of the oxidation state of Cmr on DNA-binding was assessed by EMSAs. Binding of reduced Cmr to P*cmr* was markedly enhanced compared to the oxidized protein (both diamide and H_2_O_2_-treated Cmr) (Figure 5A–C). Bio-Layer Interferometry (BLItz) allowed the kinetics of DNA-binding by Cmr to be measured in real time. Biotin-labelled P*cmr* was immobilized on streptavidin functionalized biosensor probes. Binding of Cmr to immobilized P*cmr* increases the thickness of the biological layer attached to the optical surface of the probe. This change in thickness creates an interference pattern in the reflected white light emitted by the system, which is then detected by a spectrometer. These changes were fitted to a one-site binding model to calculate rate constants for DNA-binding by Cmr. This showed that the on-rate constant for reduced (di-thiol) Cmr was ∼2-fold greater than that obtained for the oxidized (disulfide) form of Cmr (Figure 5D; Table 2). Furthermore, the dissociation rate constant for oxidized Cmr was ∼6 times greater than that of reduced Cmr (Table 2). Thus, the enhanced off-rate constant of oxidized Cmr is the major determinant of the weaker binding of the disulfide form of the protein (Figure 5D). These binding kinetics indicated that the affinity of reduced Cmr for P*cmr* (*K*_d_ = ∼2 × 10^−8^ M) was ∼10-fold greater than that for oxidized Cmr (*K*_d_ = ∼2 × 10^−7^ M) (Table 2). Replacement of one or both Cmr Cys residues by Ala locked the protein in the ‘reduced’ state resulting in enhanced DNA-binding under non-reducing conditions compared to the wild-type Cmr (Supplementary Figure S4). Thus, it was concluded that changes in the redox state of Cmr induce conformational changes that modulate its DNA-binding activity.

**Table 2.** Rate constants for binding of reduced, oxidized and nitrosated Cmr at the *cmr* promoter

Sample	*k*_a_ (M^−1^ s^−1^)^a^	*k*_d_ (s^−1^)^a^	*K*_d_ (M)^a^
Cmr reduced	1.4 × 10^5^ ±5.4 × 10^2^	2.8 × 10^−3^ ±1.9 × 10^−5^	1.9 × 10^−8^
Cmr oxidised	7.5 × 10^4^ ±1.2 × 10^3^	1.7 × 10^−2^ ±1.7 × 10^−4^	2.2 × 10^−7^
Cmr nitrosated	4.7 × 10^3^ ±3.9 × 10^2^	2.4 × 10^−3^ ±2.9 × 10^−5^	5.1 × 10^−7^

^a^Value and standard error for assays at 20°C.

### DNA-binding by Cmr is inhibited by nitrosation

**Figure 6.**
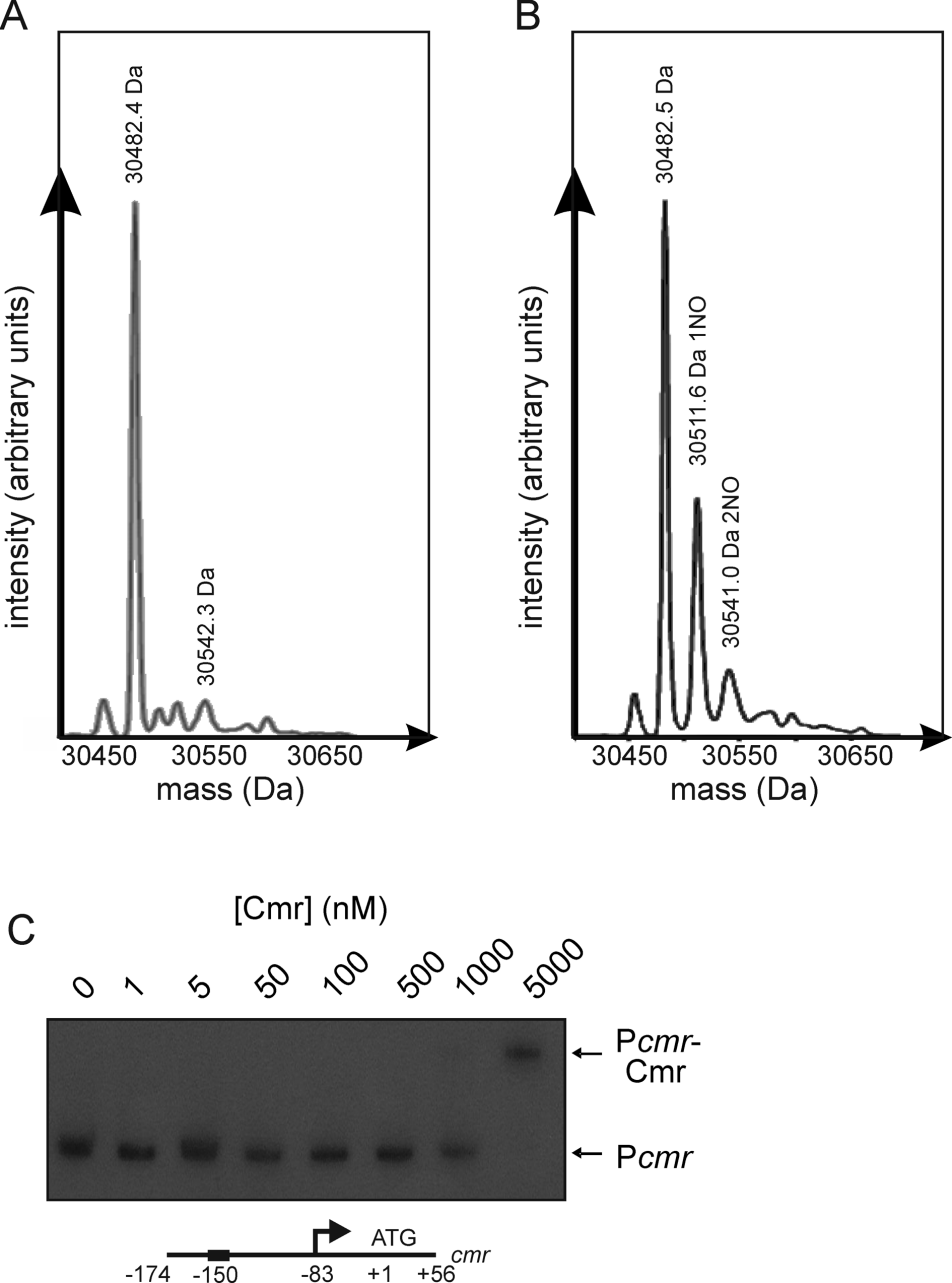
Cmr DNA-binding is inhibited by nitrosation. LC–MS of Cmr before (**A**) and after (**B**) treatment with 20-fold molar excess of GSNO for 1 h. (**C**) Radiolabelled P*cmr* was incubated with the indicated concentrations of Cmr treated with 1 mM GSNO before separation of protein–DNA complexes (P*cmr*-Cmr) from free DNA (P*cmr*) by electrophoresis. A schematic representation (not to scale) the DNA locus used in the EMSA is shown below the gel image; the location of the Cmr site (box) is indicated.

The activities of transcription factors that possess reactive thiols can be modulated by nitrosation (e.g. OxyR in bacteria and Nuclear Factor κB in eukaryotes; 31,32). LC-MS of Cmr after exposure to the nitrosating agent *S*-nitrosoglutathione (GSNO) showed peaks at 30482.5 Da (unmodified Cmr; cf. Figure 6A), 30511.6 Da (+29 Da, Cmr with one NO adduct; reaction with NO results in loss of a H atom from Cmr therefore net mass gain is 29 Da rather than the 30 Da mass of NO) and 30541.0 Da (+58.5 Da, Cmr with two NO adducts) (Figure 6B). Nitrosation of Cmr inhibited binding at P*cmr* in EMSAs (Figure 6C) and BLItz assays indicated a ∼30-fold decrease in the on-rate constant for binding at P*cmr*, without significantly altering the off-rate constant (Figure 5D; Table 2).

### Phenotypic characterization of the *M. tuberculosis cmr* mutant

**Figure 7.**
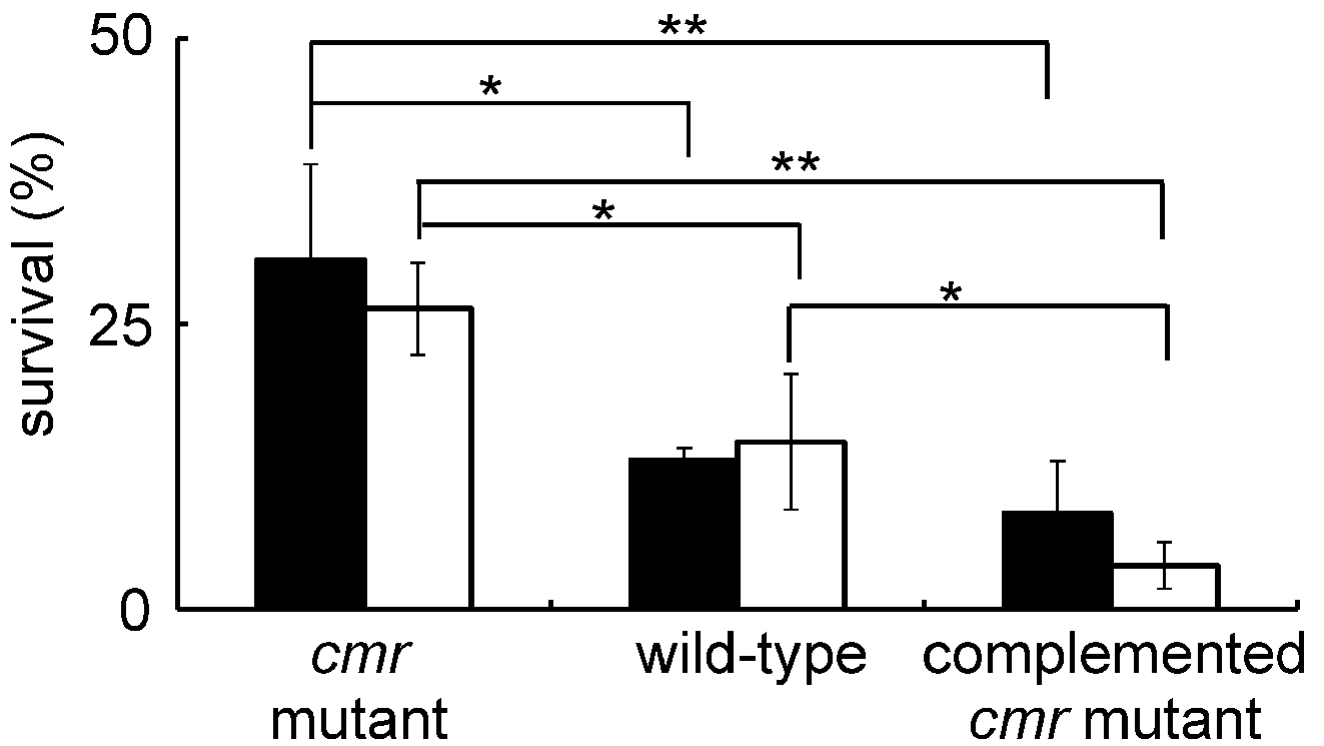
Phenotypes of the *cmr* mutant exposed to nitrosative stress. The *M. tuberculosis* H37Rv *cmr* deletion mutant, wild-type and the complemented mutant were incubated in the presence of 100 μM NO donor 6-methoxy-5-nitropyrimidin-4-yl pyrrolidine-1-carbodithioate for 24 h before assessment of survival by measurement of colony forming units (cfus). The results of two independent experiments (filled and unfilled bars) are shown. The error bars show the standard deviation from the mean (*n* = 3). Significance was determined by the Student's *t* test, ** *P* < 0.05 and * *P* < 0.1.

Modulation of the DNA-binding properties of Cmr by nitrosation suggested that it plays a role in the responses to nitrosative stress. Therefore, the phenotypes of parent, *cmr* mutant and the complemented mutant strains upon exposure of cultures to the NO donor (6-methoxy-5-nitropyrimidin-4-yl pyrrolidine-1-carbodithioate) were investigated. The viability of all three strains decreased after 24 h exposure, as judged by cfu measurements (Figure 7). However, the *cmr* mutant survived significantly better than the wild-type (*P* < 0.05, *t*-test), which in turn survived better than the complemented mutant (Figure 7). Moreover, determination of MICs for the NO donor indicated that the complemented mutant was more sensitive (0.2–0.4 μg ml^−1^) compared with the wild type and *cmr* mutant (1–2 μg ml^−1^), suggesting potential dysregulation of *cmr* in the complemented mutant. The fact that the complemented *cmr* mutant exhibited hyper-sensitivity to NO, suggested that precise control of *cmr* expression is critical for optimal function and that under- (mutant) or over- (complemented mutant) expression of *cmr* results in dysregulation. This notion was supported by qRT-PCR data showing that *cmr* was ∼2-fold over-expressed in the complemented mutant and hence there was a negative correlation between *cmr* expression and sensitivity to nitrosative stress (Supplementary Figure S5). The effect of the *cmr* deletion on the ability to survive under starvation conditions was also investigated. However, there was no significant difference in survival of the wild type strain and the deletion mutant over a 6-week period. Therefore Cmr does not appear to play a role in survival during starvation. Similar survival patterns in response to nitric oxide were previously observed in a *pknH* deletion mutant of *M. tuberculosis* indicating the complexity of regulatory pathways in this bacterium (33).

### *Mycobacterium tuberculosis cmr* is implicated in virulence

**Figure 8.**
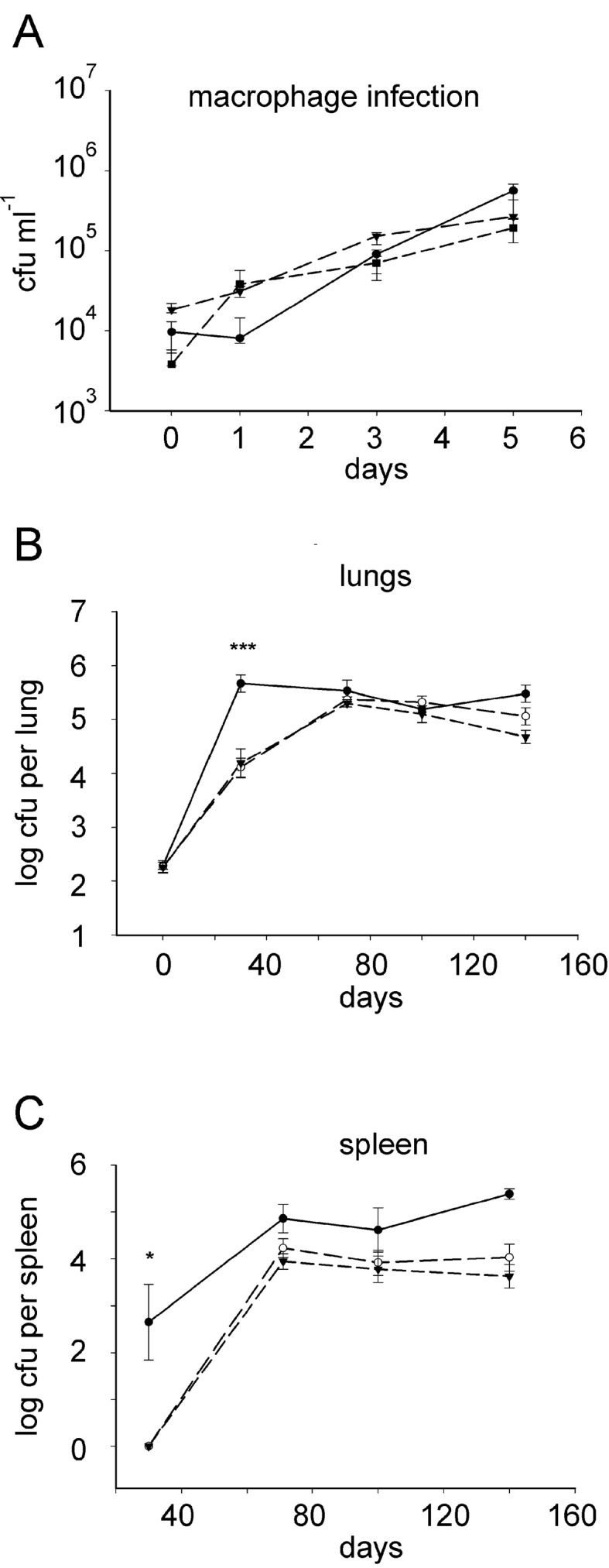
The *M. tuberculosis cmr* deletion mutant is transiently attenuated in a mouse aerosol infection model. (**A**) Growth of the *M. tuberculosis cmr* mutant is unaffected in *in vitro* infections of mouse bone marrow-derived macrophages. Survival and multiplication of *M. tuberculosis* H37Rv (WT, closed circles; solid line), the *cmr* mutant (closed inverted triangles; dashed line) and the complemented mutant strain (closed squares; dashed line) were assessed by measurement of colony forming units (cfus). The error bars show the standard error of the mean (SEM) of three replicates. (**B** and **C**) Survival and replication of *M. tuberculosis* H37Rv (closed circles; solid line), the *cmr* mutant (open circles; dashed line) and the complemented mutant strain (closed inverted triangles; dashed line) in a mouse aerosol infection model were assessed by measurement of bacterial load (cfu) in the lungs (**B**) and spleen (**C**). In each case, one of two experiments is shown. The results at each time point are the mean cfu values and the error bars show the SEM (*n* = 4–5 mice per group). Significance was determined by the Student's t test. For lungs, day 30 *cmr* mutant vs. parent *** *P* < 0.001; for spleen, day 30, *cmr* mutant versus parent * *P* < 0.05.

The growth of the mutant in Dubos medium at 37°C (μ_max_ = 0.063 h^−1^) and in mouse bone-marrow-derived macrophages was indistinguishable from that of the wild-type, in contrast to the *crp* (*rv3676*) mutant whose growth was severely attenuated (8; Figure 8A). However, in aerosol infected mice, there was a significant reduction in growth of the *cmr* mutant in both lungs and spleen 30 days after infection (Figure 8B and C). After 70 days, there was no discernible difference between the mutant and parent strains, indicating that the *cmr* mutant is transiently attenuated in the mouse model of infection. However, unlike the changes in transcriptional regulation (Table 1), it was not possible to complement the mutant phenotype using an integrating plasmid-based system in this infection model (Figure 8B and C). The possibility that the *cmr* deletion resulted in polar effects on *rv1674c-rv1672c* was considered unlikely because the transcriptomic data indicated that expression of these genes was unaltered in the *cmr* mutant strain. Therefore, the genome sequences of the parent and *cmr* mutant strains were obtained. Sequencing revealed the presence of four gene deletions (*rv0796, rv3184, rv3185* and *rv3326*) associated with transposases in both strains, as well as the intended *cmr* deletion in the mutant strain. The *rv0796, rv3184* and *rv3185* genes are deleted in some *M. tuberculosis* clinical isolates and therefore are unlikely to be necessary for virulence (Supplementary Table S3). In addition to these deletions, the *cmr* mutant had five single nucleotide polymorphisms (SNPs) compared to the parent. Four of these SNPs were unlikely to have any detrimental effect on the mutant because the genes affected are not essential for growth (*fadE6*: SNP, P7L; *rv2323*: SNP, L202P; *rv3331*: SNP, P423T) or are found in other virulent *M. tuberculosis* strains (*prpD*; SNP, R9P). Therefore, it was concluded that individually none of these differences are likely to account for the observed attenuation of the *cmr* mutant (further details are provided in Supplementary Table S2). However, it is possible that, in combination, these genomic changes could account for the inability to complement the *cmr* mutant in the infection model; an alternative explanation is that the amount of Cmr in the bacterium has to be tightly controlled (see above). The latter explanation would be consistent with the observed high-affinity binding of Cmr at P*cmr* and consequent auto-regulation and the fact that over-expression of *cmr* from an *hsp60* constitutive promoter resulted in inhibition of *M. tuberculosis* growth (Figure 2; Table 2; not shown).

**Figure 9.**
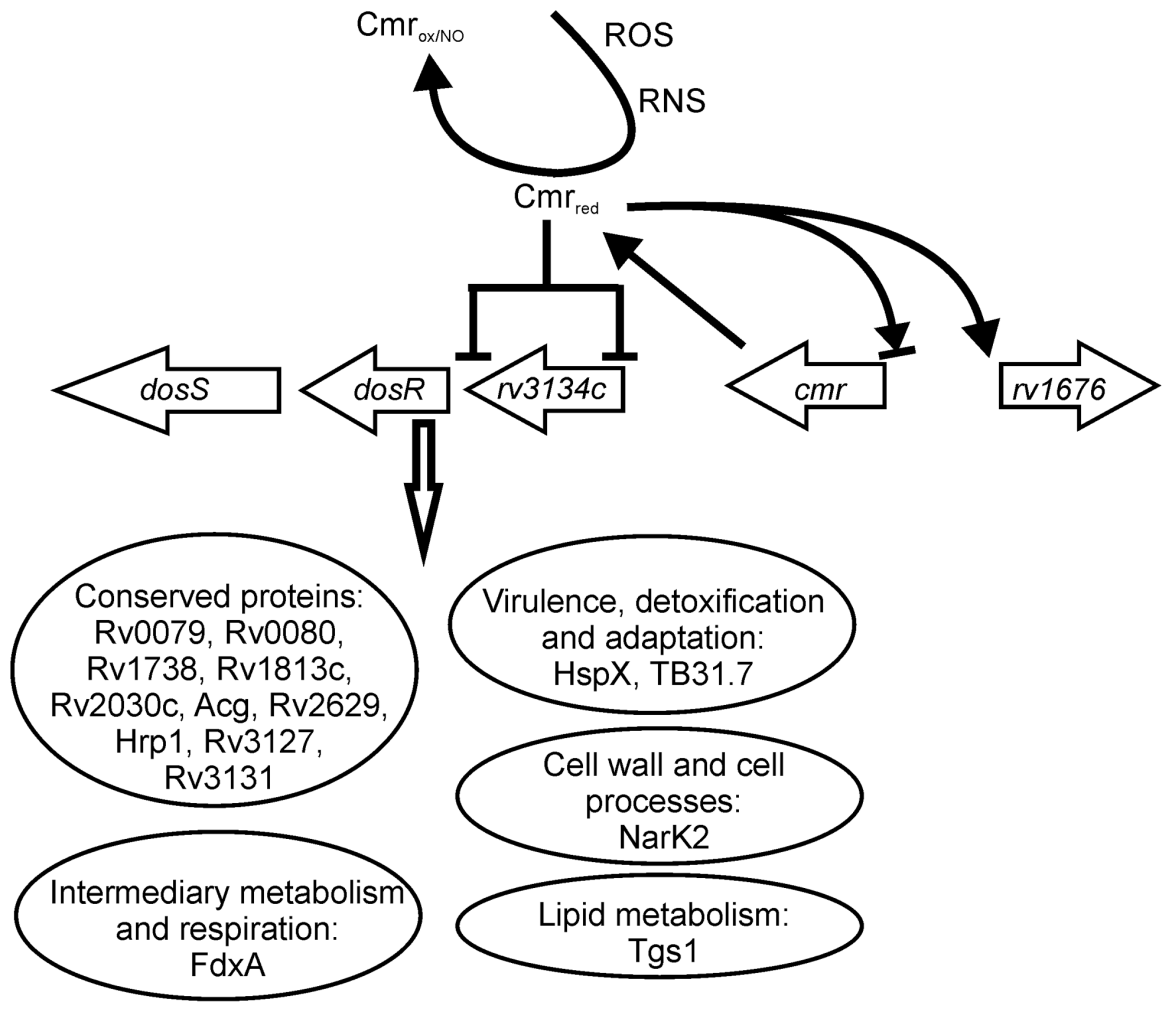
Working model for the role of Cmr in regulation of the Dos-regulon in response to oxidative and nitrosative stresses. Under non-stressed aerobic growth conditions Cmr exhibits high affinity site-specific DNA-binding (Cmr_red_) and acts as a dual regulator of its own expression, activates expression of the divergent *rv1676* gene and represses expression of DosR. Nitrosation (and to a lesser extent oxidation) of Cmr (Cmr_ox/NO_) results in de-repression of *dosR*, thereby activating expression of a subset of the DosR-regulon (enclosed in ellipses).

## CONCLUDING REMARKS

Biochemical and molecular genetic analyses of the *M. tuberculosis* transcription factor Cmr indicated that site-specific binding to DNA sequences resembling the palindrome TGTCAGCGTGCTGACA (Figure 2) was severely impaired upon exposure to nitrosative stress via nitrosation of two cysteine residues (Figures 5 and 6). DNA-binding by Cmr was also impaired, but less severely, when the same cysteine residues were linked by formation of an intramolecular disulfide bond, suggesting that nitrosative stress is the major modulator of Cmr activity (Figures 4 and 5). Transcriptional profiling and EMSAs indicated that Cmr repressed major components of the DosR-regulon by binding at two promoters in the *rv3134c-dosR-dosS* operon (Table 1; Figure 3). De-repression of the DosR-regulon in the *cmr* mutant potentially prepares the bacterium for the challenges posed by nitrosative stress, providing a plausible explanation for the observed enhanced resistance of the mutant compared to the wild-type (Figure 7). It is notable that the W/Beijing strains of *M. tuberculosis*, which are attributed with properties of enhanced transmission, virulence and drug-resistance, constitutively over-express the DosR-regulon in laboratory cultures (34). It is thought that the up-regulation of the DosR-regulon prior to exposure to inducing signals (usually associated with inhibition of respiration and replication e.g. hypoxia and NO) pre-conditions W/Beijing strains to survive the assaults of the host immune system (35). This interpretation is consistent with the observation that the *cmr* mutant exhibits enhanced survival under nitrosative stress conditions (Figure 7). Interestingly, the *rv1671* and *rv1672c-1674c* genes (Region of Difference 150) that are located immediately adjacent to *cmr* were found to be deleted in six strains of the East Asia clade of W/Beijing strains (36). It is possible that these deletions modulate the expression of *cmr*, thereby contributing to the over-expression of the DosR-regulon. However, de-repression of the DosR-regulon only partially accounts for the observations reported here. Whilst the ‘stress-ready’ state of the *M. tuberculosis cmr* mutant provides a survival advantage when challenged with nitric oxide donors in laboratory cultures (Figure 7), it is neutral for survival in cultured macrophages and results in transient attenuation in an aerosol model of infection (Figure 8). This suggests that Cmr-mediated gene regulation is complex and its influence over *M. tuberculosis* stress responses extends beyond de-repression of the DosR-regulon, as indicated by the ChIP-seq data reported by Ranganathan *et al.* for *M. bovis* BCG (13). Moreover, Cmr-mediated gene expression could be further complicated by its capacity to respond to both oxidation and nitrosation. *Escherichia coli* OxyR is a transcription factor that can transduce different redox signals into bespoke transcriptional responses via the altered DNA-binding affinities and cooperative behaviours of the oxidized and nitrosated forms of the protein (31). The similarities in signal perception and effects on DNA-binding affinity (high affinity binding for reduced Cmr that was lowered for the disulfide form and further impaired for the nitrosated form) suggest that Cmr could be functionally equivalent to OxyR (Figure 9). Further work to explore the breadth of the *M. tuberculosis* Cmr regulon should allow this suggestion to be tested.

## Supplementary Material

Supplementary Data
