# Supplementary material for: Cmr is a redox-responsive regulator of DosR that contributes to M. tuberculosis virulence
Source: Nucleic Acids Res. 2017 May 8;45(11):6600–12. doi: 10.1093/nar/gkx406 (PMC5499769; doi:10.1093/nar/gkx406)
Supplement: Supplementary Data [file gkx406_Supp.zip › nar-02671-v-2016-File011.pdf]

Supplementary information

Cmr is a redox-responsive regulator of DosR that contributes to *M. tuberculosis* virulence

Laura J. Smith[1,2*], Aleksandra Bochkareva[1], Matthew D. Rolfe[1], Debbie M. Hunt[3], Christina Kahramanoglou[3], Yvonne Braun[3], Angela Rodgers[3], Alix Blockley[3], Stephen Coade[3], Kathryn E.A. Lougheed[3], Nor Azian Hafneh[4], Sarah M. Glenn[4], Jason C. Crack[5], Nick E. LeBrun[5], José W. Saldanha[6], Vadim Makarov[7], Irene Nobeli[8], Kristine Arnvig[9], Galina V. Mukamolova[4], Roger S. Buxton[3] and Jeffrey Green[1]

[1]Molecular Biology and Biotechnology, University of Sheffield, Sheffield, S10 2TN, UK; [2]School of Pharmacy, De Montfort University, Leicester, LE1 9BH, UK; [3]Division of Mycobacterial Research, MRC National Institute for Medical Research, Mill Hill, London, NW7 1AA, UK; [4]Department of Infection, Immunity and Inflammation, University of Leicester, University Road, Leicester, LE1 9HN, UK, [5]Centre for Molecular and Structural Biochemistry, School of Chemistry, University of East Anglia, Norwich, NR4 7TJ, UK; [6]Division of Mathematical Biology, MRC National Institute for Medical Research, Mill Hill, London, NW7 1AA, UK; [7]A. N. Bach Institute of Biochemistry, Russian Academy of Sciences, Moscow, Russia; [8]Institute of Structural and Molecular Biology, Department of Biological Sciences, Birkbeck, University of London, Malet Street, London, WC1E 7HX, UK; [9]Institute for Structural and Molecular Biology, University College London, London, WC1E 6BT, UK

Contents

Supplementary methods

Determination of the oligomeric state of Cmr

DNA fragments used in EMSAs

DNA fragments used as templates for *in vitro* transcription reactions

Attempted reconstitution of Cmr with heme

Quantitative-RT-PCR (qRT-PCR)

Supplementary Figures

Figure S1. Cmr is dimeric.

Figure S2: Identification of *cmr* transcript starts *in vivo* and *in vitro* and the effect of cAMP on transcription *in vitro*.

Figure S3: DNA sequences upstream of the *rv3134c* and *rv3133c* (*dosR*) open reading frames.

Figure S4. Replacement of Cmr Cys residues enhances DNA-binding under non-reducing conditions.

Figure S5. Expression of *cmr* in the parent and complemented *cmr* mutant strains of *M. tuberculosis*.

Supplementary Tables

Table S1. List of strains and plasmids used in this study

Table S2. Excel spreadsheet of gene expression data (provided as a separate file)

Table S3. Comparison of the genome sequences of the *M. tuberculosis* H37Rv (parent) and the *M. tuberculosis* H37Rv *rv1675c* deletion (mutant) strain

Supplementary References

Supplementary methods

Determination of the oligomeric state of Cmr

Gel filtration was carried out using a calibrated Superdex column, equilibrated with 50 mM sodium phosphate buffer (pH 7.4) and 150 mM NaCl. Cmr samples (~1 mg ml^{-1}) were treated with the indicated reagent before analysis and compared to a standard curve using proteins of known molecular mass. Crosslinking was carried out using purified Cmr (0.8 mg ml^{-1}; 26.3 μM monomer). Samples were incubated for 60 min at 20°C with a 20-fold molar excess of dimethyl 3,3´-dithiobispropionimidate (DTBP) an imidoester cross-linker. DTBP was solubilized in 0.2 M triethanolamine buffer (pH 8.0) before the addition of Tris-HCl buffer pH 8.3 (final concentration 20 mM) to stop the reaction. Samples were then analyzed on SDS-PAGE (polyacrylamide gel electrophoresis).

DNA fragments used in EMSAs

Details of the DNA sequences used in the EMSAs reported here are provided below. Coding regions in black; non-coding regions in red, transcript starts in bold upper case (P*cmr*, this work; P*groEL2*, Stapleton *et al.*, (1); others from Cortes *et al.*, (2)), start codon for target gene underlined, start codon for divergent gene double underlined.

1. P*groEL2*

375 bp fragment ligated into pCR4Blunt TOPO – released by *Hin*dIII and *Xba*I from p2126

gcggccgaccatttacgggtcttgttgtcgttggcggtcatgggccgaacatactcacccggatcgga
gggccgaggacacggtcgaacgagggcatgacccggtgcggggcttcttgcactcggcataggcgag
tgctaagaataacgttg**G**cactcgcgaccggtgagtgctaggtcgggacggtgaggccaggcccgtcg
tcgcagcgagtggcagcgaggacaacttgagccgtccgtcgcgggcactgcgcccggccagcgtaagt
agcggggttgccgtcacccggtgaccccgtttcatccccgatccggaggaatcacttcgcaatggcc
aagacaattgcgtacgacgaagaggcccgtcgcg

2. P*cmr*

230 bp fragment ligated into pGemTEasy – released by *Eco*RI from pGS2462

ccgccaagaatcggtttgtcagcgtgctgacagagt**G**cctggggcgcagaacgggcttgcgtgttctc
ttcgatcgactaatttcccattc**A**gggcaagccaacccgcacgcaatccacgcgcgctgacaattggc
agcacgatgacggcatcgcctgctctcaaggaggggccatggcagatcggtcggtgcgcccgctgcgg
catctcgttcatgcggtgactggggg

3. P*rv2007c*

160 bp fragment ligated into pCR4Blunt TOPO – released by *Hin*dIII and *Xba*I from p2365

gagcgtgaatcccgctggcggtcgggtgaaccgccccggttttcttgcaccccgcgtcgacgtgccag
tgacgaacttgacgaataaggcctttggtcctttccggtaggggtctttggataggcgcgatcctcgg
catcgggccggtagcttgccgt**T**tgtg

4. P*rv2032*

201 bp fragment ligated into pCR4Blunt TOPO – released by *Hin*dIII and *Xba*I from p2366

tttgatgcctcctaatcgatggaaacggatgcctttgatccgaccagcccatcgtggccagggctagg
gacagaagtccccgaagcgcgggccatttgtccgcgcccgtcggtgatccacttggggaccattgacc
ctgttgtctgccaaccgccgttcagaaagatcggggt**G**atatcgaacagcggaggttgatcatgc

5. P*rv3133c*

428 bp fragment ligated into pCR4Blunt TOPO – released by *Hin*dIII and *Xba*I from p2367

cgcggaggtggacaatggtgtggtgctgcggcacgcattcgaggaggccaggctgcgcggagttccgc
tgcgggccgtggctgtccacgctgctgaaacacccgatgacgtcgaacagggcagccggttggcgcat
gtacacctgagccgtcggctcgcccactggacccggctctaccccgaggtgcgggtggatcgggccat
cgccggcggcagtgcgtgccgtcatctggccgccaacgcaaagccgggtcagctgttcgtcgcggact
cacactccgcgcacgaattgtgcggtgcataccagcccggatgcgccgtacttacggtacgcagtgcc
aacttgtagggagcggat**C**ttgggagtggtgccctggtggtaaaggtcttcttggtcgatgaccacga
ggtggtgcgtcgtggtctgg

6. P*rv3134c*

584 bp fragment ligated into pCR4Blunt TOPO – released by *Hin*dIII and *Xba*I from p2344

tactccgcaggcccctaacgtcgattgcgcacccagcccaattcggacctcggtgtgggcatgcggga
gacggcagtggctccccccatggtcacgagcggctccgagtgggctccgctcgggcgcaggccggctg
atacttgttgaccagtgcggcaactcgtgccgcggccagtgtacaactgggcgggcaggttgtatggc
agtcgactgacggcggatcggagattgatcgacgccgtgccggccgcatcgagatgcgctctcacagc
gcggctatgcccaaatattctgtgggtcaagcgatatgcagccgatggacggccgctggttcggcagc
tgtcggcaactgtaagccatttctgggactttgctgtgaaaagctgggcgatggttgtggacctggac
gagccacccgtgcgataggtgagattcattctcgccctgacgggttgcgtctgtcatcggtcgataag
gactaacggccctcaggtggggaccaacgcccctgggagatagcggtccccgccagtaacgtaccgct
Gaaccgacgggatgtatccgccccagcgaaggagacggcgatg

DNA fragments used as templates for *in vitro* transcription reactions

DNA sequences used in the *in vitro* transcription reactions are provided below. For the *cmr* templates the transcript starts (TSS_1 and TSS_2; Fig. 2E) are shown in bold font and the *rv1676* transcript start is underlined.

1. *rrnAP3*

TAGAGCAATTCGAACGGGTATGCTGTTAGGCGACGGTCACCTATGGATATCTATGGATGACC
GAACCTGGTCTTGACTCCATTGCCGGATTTGTATTAGACTGGCAGGGTTGCCCCGAAGCGGG
CGGAAACAAGCAAGCGTGTTGTTTGAGAACTCAATAGTGTGTTTGGTGGTTTCACAGCAATT
CTCTA

2. *cmr-rv1676* (282 bp)

TAGAGAATTCGTCCACCGGTTGATCGTTGATGTCCTGCAGACGGCGAGTCGGCACGGTCGCA
CCGCGGGCCAATCGAGTAGGCACCCCAATACCATACC**G**ATGGCAAAACTCCGCCAAGAATCG
GTTTATTAGCGTGCTAATAGAGTGCCTGGGGCGCAGAACGGGCTTGCGTGTTCTCTTCGATC
GACTAATTTCCCATTCAGGGC**A**AGCCAACCCGCACGCAATCCACGCGCGCTGACAATTGGCA
GCACGATGACGGCATCGCCTGCTCGAATTCTCTA

3. *cmr-rv1676trunc1* (234 bp)

GCACGGTCGCACCGCGGGCCAATCGAGTAGGCACCCCAATACCATACC**G**ATGGCAAAACTCC
GCCAAGAATCGGTTTGTCAGCGTGCTGACAGAGTGCCTGGGGCGCAGAACGGGCTTGCGTGT
TCTCTTCGATCGACTAATTTCCCATTC**A**GGGCAAGCCAACCCGCACGCAATCCACGCGCGCT
GACAATTGGCAGCACGATGACGGCATCGCCTGCTCTCAAGGAGGGGCC

4. *cmr-rv1676trunc2* (171 bp)

CCGCCAAGAATCGGTTTGTCAGCGTGCTGACAGAGTGCCTGGGGCGCAGAACGGGCTTGCGT
GTTCTCTTCGATCGACTAATTTCCCATTC**A**GGGCAAGCCAACCCGCACGCAATCCACGCGCG
CTGACAATTGGCAGCACGATGACGGCATCGCCTGCTCGAATTCTCTA

Attempted reconstitution of Cmr with heme

A 50 mM stock solution of hemin was prepared in 100 mM KOH and was used to prepare a working solution of 1 mM in 20 mM sodium phosphate, pH 7.4 containing 0.5 M NaCl. The concentration of the solution was verified spectrophotometrically (ε = 58400 M^{-1} cm^{-1} at 385 nm). A solution of Cmr (9.6 μM) was titrated with hemin (0.5 μM aliquots) and absorbance

spectra were recorded after 2 min incubation at 20°C. As a control, the same concentration of the non-heme-binding protein Crp^{Mt} (Rv3676) was subjected to similar titrations.

Quantitative-RT-PCR (qRT-PCR)

RNA was isolated from 9 ml of early logarithmic phase (OD_{580} 0.15-0.2) *M. tuberculosis* H37Rv cultures using the TRIZOL method (3). DNA contamination was removed using the TURBO DNA-*free*™ kit (Ambion, Life Technologies). After purification, total RNA was used for cDNA synthesis, using Superscript™ II Reverse Transcriptase (Invitrogen), according to the manufacturer's instructions. qRTPCR was performed using primers specific for 16S *rRNA* gene, Myco16sF (5´-GAAACTGGGTCTAATACCG-3´) and Myco16sR (5´-ATCTCAGTCCCAGTGTGG-3´) and *cmr* gene specific primers, RT1675F (5´-AATCCGTGTCGCACAATCCA-3´) and RT1675R (5´-GTGACGATGTGTCGGCATTG-3´). qRT-PCR experiments were carried out in a Corbett Rotor Gene 6000 real time thermocycler, using a previously published protocol (4). Three technical replicates were performed for each experimental sample. Copy numbers of *cmr* transcripts per 1 μg of RNA were calculated and normalised to 16S *rRNA* expression.

Supplementary Figures

(A)

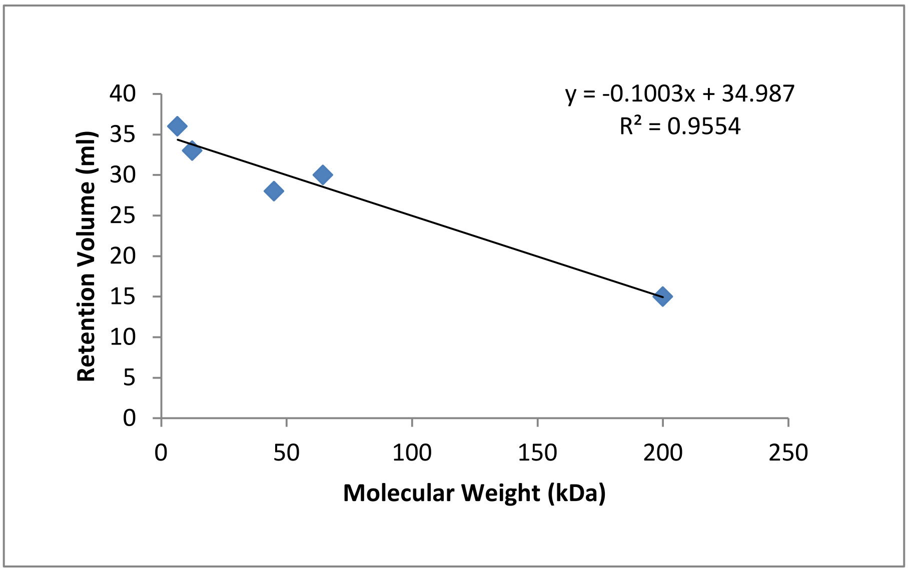

(B)

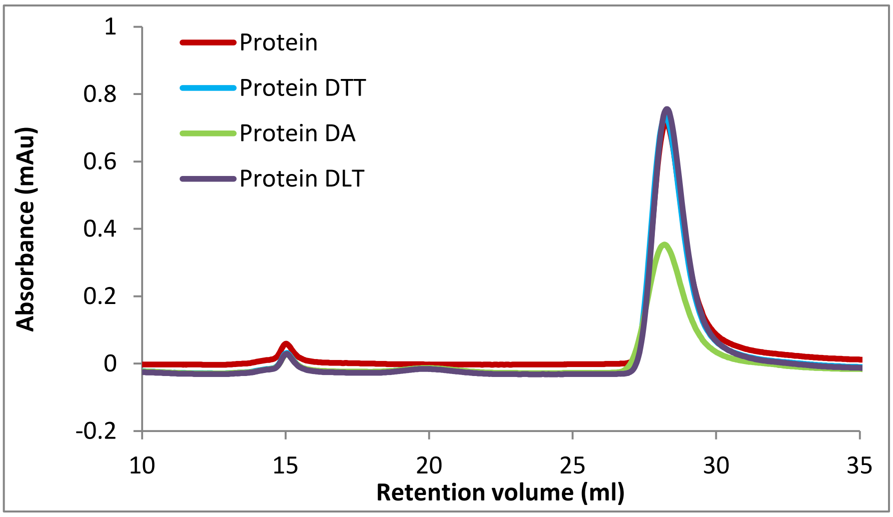

(C)

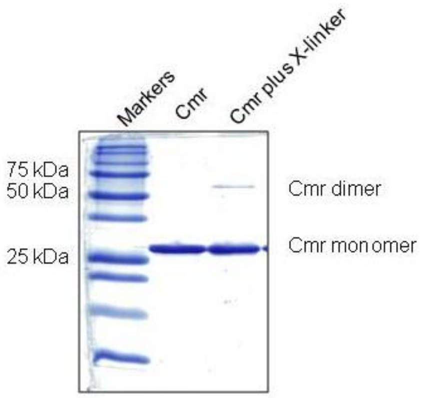

Figure S1. Cmr is dimeric. (A) Calibration curve for Superdex size exclusion chromatography equilibrated with 20 mM sodium phosphate, pH 7.4 containing 0.15 M NaCl. Standards were: Blue dextran (200 kDa); hemoglobin (64.5 kDa); ovalbumin (43 kDa); cytochrome *c* (12.4 kDa); and aprotinin (6.5 kDa). (B) Elution profiles for Cmr as prepared (Protein, red trace), reduced (dithiothreitol, 1 mM, Protein DTT, blue trace), oxidized (diamide, 1 mM, Protein DA, green trace) and a threitol (1 mM) control (Protein DLT, purple trace). All profiles were consistent with Cmr eluting as a dimer (~70 kDa). (C) Chemical cross-linking of Cmr with dimethyl 3,3´-dithiobispropionimidate (DTBP). Separation by SDS-PAGE (denaturing) showed the presence of species of molecular weight ~30 kDa, equivalent to uncross-linked Cmr monomer and ~60 kDa, equivalent to a cross-linked Cmr dimer. The samples shown are aliquots of Cmr (0.8 mg ml^{-1}) without treatment, after 60 min incubation with a 20-fold molar excess of DTBP (Cmr plus X-linker).

(A)

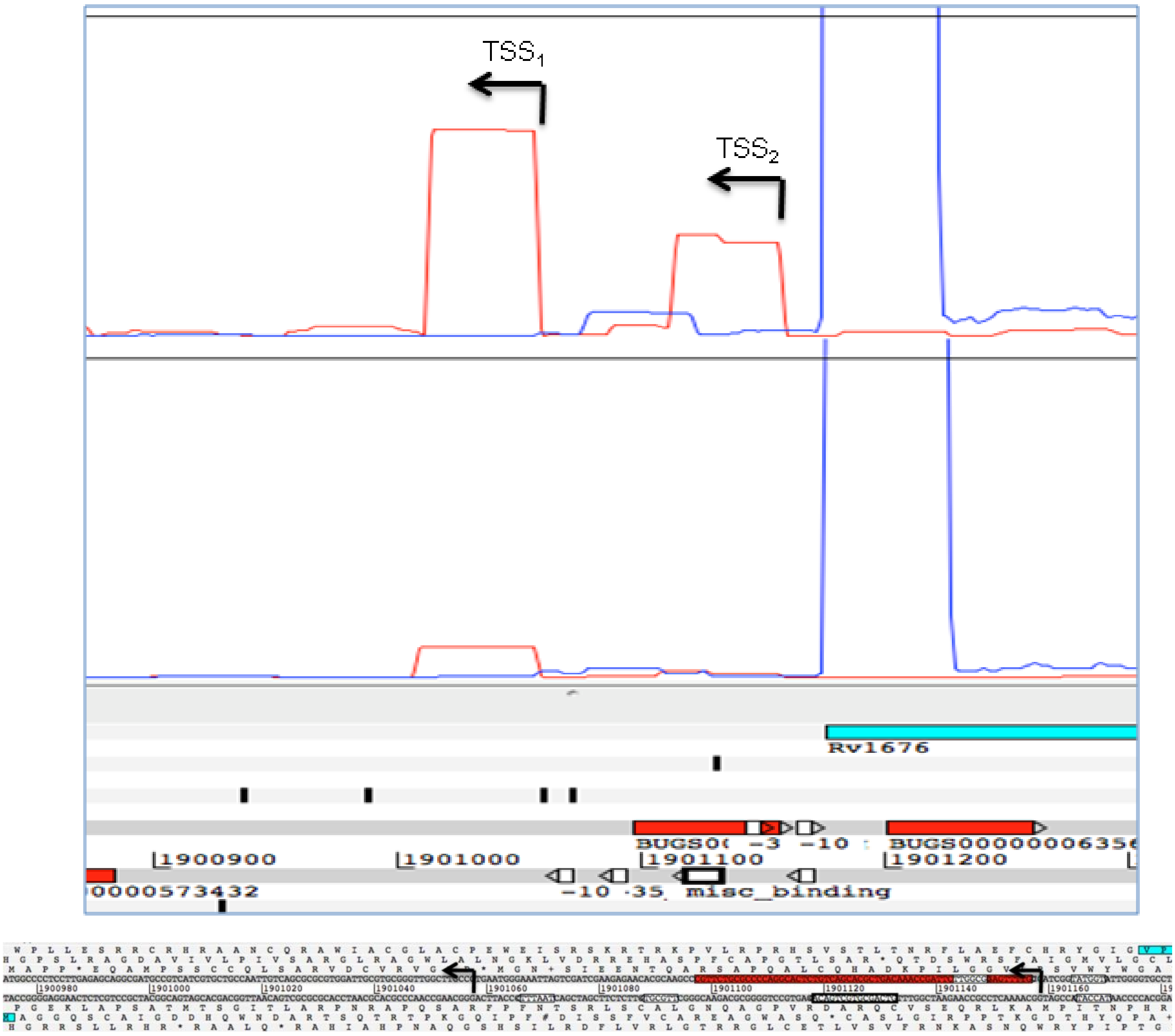

TSS1
TSS2
Rv1676
BUGS0(
-3
-10
BUGS0000000635
1900900
1901000
1901100
1901200
00000573432
-10
-35
misc_binding

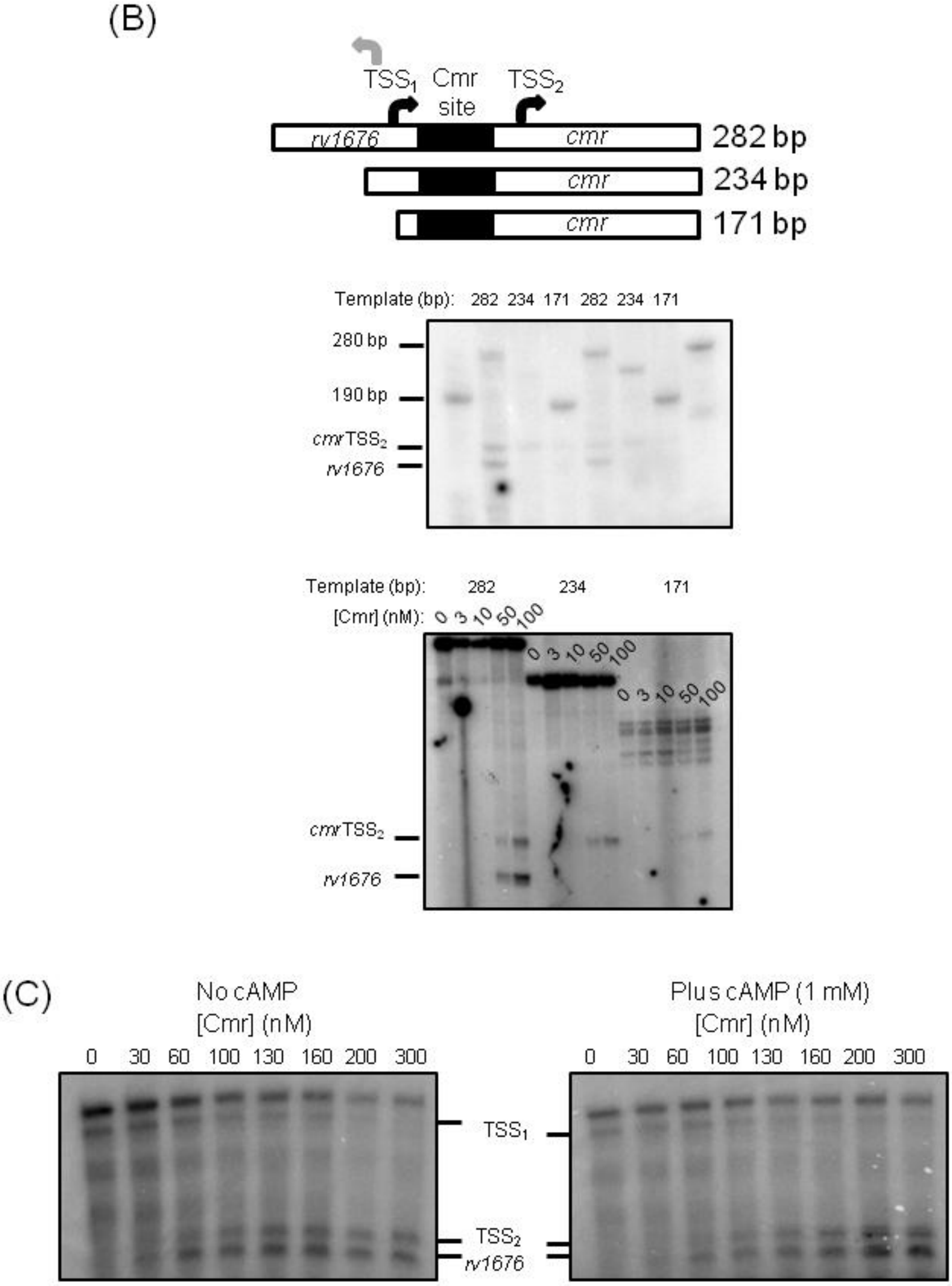

Figure S2: Identification of *cmr* transcript starts *in vivo* and *in vitro* and the effect of cAMP on transcription *in vitro*. (A) Artemis traces showing two transcript start sites (arrowed, TSS) upstream

of the *cmr* (*rv1675c*) coding region (2). The red line indicates the transcription in the reverse direction and the blue line for the forward direction. The lower panel shows the DNA and amino acid sequences for this region. The initial Met (M) of Cmr is highlighted in pale blue on the left and the beginning of the Rv1676 open reading frame is similarly highlighted on the right. (B) Identification of the Cmr-activated *rv1676* transcript. The diagram (not to scale) show the different templates used in the transcription reactions. The black arrows indicate the *cmr* transcript starts; the grey arrow indicates the *rv1676* transcript start; the black rectangle indicates the Cmr binding site. Upper autoradiograph: Different fragments of DNA containing the *rv1675c-rv1676* intergenic region were incubated with *M. smegmatis* RNA polymerase in the presence of 5 nM (left lanes marked 282, 234 and 171) or 1 nM (right lanes marked 282, 234 and 171) Cmr protein; the numbers indicate the length of the DNA templates used shown diagrammatically above the autoradiographs. The sequences of the DNA templates used are provided in *Supplementary methods* section (see above). Based on the data shown in Fig. 2E, the 282 bp, 234 bp and 171 bp templates in the presence of Cmr should all yield the *cmr* transcript of ~80 bases. The *rv1676* transcript should be present (~80 bases) when the 282 bp template is used, but would be undetectable (37 bases and absent) for the shorter templates. Thus, using templates with shorter DNA sequences downstream of the *rv1676* transcript start it was possible to assign the Cmr-activated *cmr* transcript. Transcription reactions were carried out as described in the *Materials and Methods*. The *cmr* (TSS_2) and *rv1676* transcripts are indicated. Transcripts of know sizes (280 bases and 190 bases) are shown in the outermost lanes of the middle autoradiograph to calibrate the gel. Lower autoradiograph: in this experiment the different DNA templates were subject to *in vitro* transcription in the presence of increasing concentrations of Cmr as indicated above each lane. (C) Cyclic AMP does not alter Cmr-regulated transcription from the *cmr-rv1676* intergenic region in *in vitro* transcription reactions. The locations of transcripts assigned to *cmr* (TSS_1 and TSS_2) and *rv1676* are indicated.

(A)

tgggcgatggttgtggacctggacgagccacccgtgcgataggtgagattcat

tctcgccctgacgggttgcgtct**GTCA**t**C**ggt**C**gataaggactaacggccctc

aggtggggaccaacgcccctgggagatagcg**GTC**cc**C**gcca**G**taacgtaccgc

t<u>g</u>aaccgacgggatgtatccgccccagcgaaggagacggcgATG

(B)

accccgaggtgcgggtggatcgggccatcgccggcggcagtgcgtgcc**GTCA**t

Ctg**G**ccgc**C**aacgcaaagccgg**GTCAGC**tgttc**G**t**C**gcggactcacactccgc

gcacgaattgtgcggtgcataccagcccggatgcgccgtacttacggtacgca

gtgccaacttgtagggagcggat<u>c</u>ttgggagtggtgccctgGTG

Figure S3: DNA sequences upstream of the *rv3134c* and *rv3133c* (*dosR*) open reading frames. DNA sequences (200 bp) upstream of (A) the *rv3134c* open reading frame (ATG start codon) and (B) the *rv3133c* open reading frame (GTG start codon). Potential Cmr binding sites are indicated in bold type face with bases that match the consensus sequence (GTCAGCnnGTGAC; identified by ChIP-seq analyses and confirmed by mutation of this site at the *cmr* promoter) shown in upper case. The start codons are shown in upper case and the transcript start sites are underlined (2).

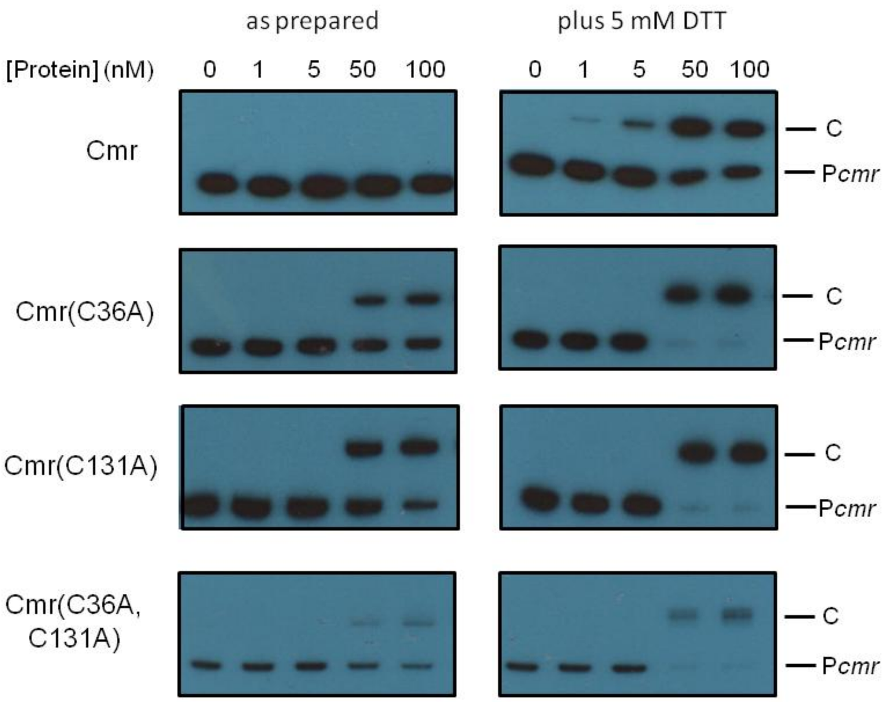

Figure S4. Replacement of Cmr Cys residues enhances DNA-binding under non-reducing conditions. Cmr variants with either one or both Cys residues replaced by Ala were created by site-directed mutagenesis. The corresponding proteins were incubated with radiolabelled *cmr-rv1676* intergenic region DNA (P*cmr*) without (as prepared) or with dithiothreitol (plus 5 mM DTT) and the protein-DNA complexes (C) formed were separated by electrophoresis and visualized by autoradiography. The final concentrations of the indicated Cmr proteins used in the EMSA are indicated above the gel images.

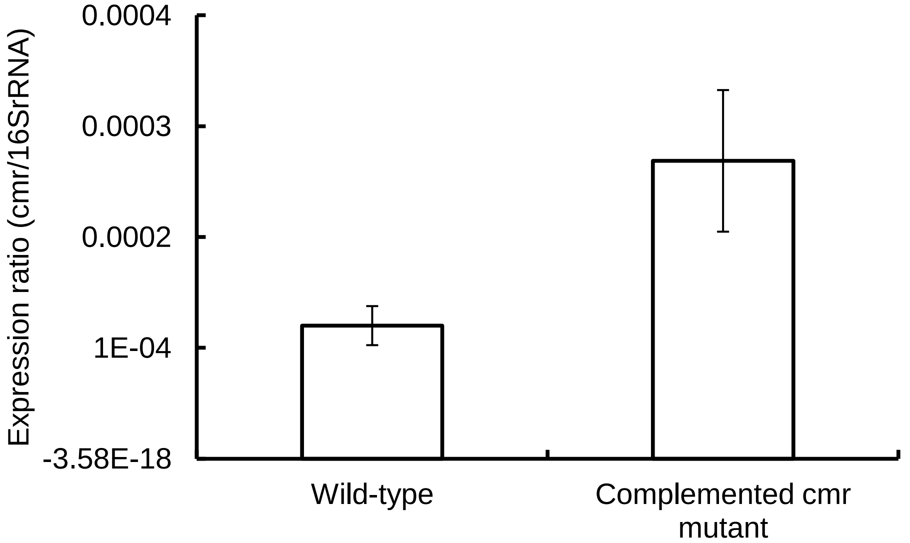

Figure S5. Expression of *cmr* in the parent and complemented *cmr* mutant strains of *M. tuberculosis*. Total RNA was isolated from early logarithmic phase (OD_{580} 0.15-0.2) *M. tuberculosis* H37Rv cultures (wild-type and complemented *cmr* mutant) and *cmr* cDNA was synthesized as described in *Supplementary methods*. Copy numbers of *cmr* transcripts per 1 μg of RNA were calculated and normalized to 16S *rRNA* expression (the mean values and standard deviations are shown). The data suggest a 2.2-fold increase in expression of *cmr* in the complemented mutant compared to the wild-type strain.

Supplementary Tables

Table S1: List of strains and plasmids used in this study.

**Strain**	**Relevant characteristics**	**Source or Reference**
BL21 λ(DE3)	*Escherichia coli* BL21 λ(DE3) lysogen carrying a copy of the T7 RNA polymerase under the control of the IPTG-inducible *lacUV5* promoter	Novagen
JRG2357	DH5α *ΔlacZYA-argF, lacZΔM15*	Invitrogen
Mycobacterium smegmatis mc^2 155	RNAP production	(5)
Mycobacterium tuberculosis H37Rv	Wild-type virulent strain	Lab strain
Mycobacterium tuberculosis Δ*cmr*	Deletion mutant of *cmr*	This work
Mycobacterium tuberculosis cmr complement	*M. tuberculosis* Δ*cmr/cmr*	This work

**Plasmid**	**Relevant characteristics**	**Source or Reference**
pGS2103	pET28a-*cmr*; Kan^R	This work
p2126	pCR4Blunt-TOPO-P*groEL2*; Kan^R and	D. M. Hunt, NIMR

	Amp^R	
p2365	pCR4Blunt-TOPO-P*rv2007c*; Kan^R	D. M. Hunt, NIMR
p2366	pCR4Blunt-TOPO-P*rv2032*; Kan^R	D. M. Hunt, NIMR
p2367	pCR4Blunt-TOPO-P*rv3133c*; Kan^R	D. M. Hunt, NIMR
p2344	pCR4Blunt-TOPO-P*rv3134c*; Kan^R	D. M. Hunt, NIMR
pGS2462	pGEM-tEASY-P*cmr*	This work
pGS2531	pGEM-tEASY-P*cmr* with altered bases in the palindromic binding site	This work

***M. tuberculosis* shuttle plasmids**	**Relevant characteristics**	**Source or Reference**
p2NIL	Suicide gene delivery vector, *oriE*, *kan*	T. Parish (6)
pGOAL17	P_{hsp60}–*sacB*, P_{Ag85a} –*lacZ* marker gene cassette, *amp*, *oriE*	T. Parish (6)
P2NIL:1675c.17	Delivery plasmid for deletion of *cmr* gene; Kan^R	This work
pKP186	Integrase-negative derivative of the integrating vector pMV306	K.G. Papavinasasundaram (unpublished)

pBS-int	Integrase-positive plasmid lacking a *M. tuberculosis* origin of replication	B. Springer (unpublished)

* AmpR, ampicillin resistant; Kan^R, kanamycin resistant

Table S3. Comparison of the genome sequences of the *M. tuberculosis* H37Rv (parent) and the *M. tuberculosis* H37Rv *cmr* deletion (mutant) strain.

Strain	Gene	Comment	Reference
Mutant	*fadE6, rv0271*	SNP: P7L, the first residue in the domain acyl-CoA-dehydrogenase domain; non-essential for growth; pseudogene in *M. leprae*	(7-9)
Mutant	*rv0796*	Deleted (14 bp remaining); transposase; deleted in clinical isolates	(10)
Mutant	*rv1130, prpD*	SNP: R9P; the same SNP is found in *M. tuberculosis* BTB04-172 (monoisolate); R9H in *M. tuberculosis* TKK-01-0058<br><br>Erdman *prpDC* mutant is unable to grow on propionate; growth is severely impaired in non-activated murine bone marrow-derived macrophages; growth and persistence in the lung and spleen is comparable to wild-type; absent in *M. leprae*	(7,8, This work)
Mutant	*rv1675c, cmr*	Deleted (12 bp remaining)	This work
Parent	*rv1960, parD1*	SNP: R51G; ParD antitotxin; non-essential for growth; absent in *M. leprae*; SNPs were found in *M. tuberculosis* strains: M1V; A78P; G82A	(7,8, This work)
Mutant	*rv2323, dhaH*	SNP: L202P; multiple SNPs in *M. tuberculosis* strains, R30H; V72I, A120T, H201Y, T213A; S222N; non-essential amidinotransferase; pseudogene in *M. leprae*	(7,8, This work)
Parent	*rv2962*	SNP: T234I; other SNP in database H233Q; in *M. leprae* T234Q; non-essential uridine 5'-	(7,8)

		diphospho-glucuronosyltransferase	
Mutant	*rv2984, ppk1*	SNP: Q476R in phospholipase domain; essential for *in vitro* growth; impaired stress survival; down-regulation results in impaired survival in macrophages	(7,8,11)
Mutant	*rv3184*	Deleted (12 bp remaining); transposase; deleted in clinical isolates	(7,8,10)
Mutant	*rv3185*	Deleted (0 bp remaining); transposase; deleted in clinical isolates	(7,8,10)
Mutant	*rv3326*	Deleted (8 bp remaining); transposase	(7)
Mutant	*rv3331, sugI*	SNP: P423T; other SNP in database P423L (most strains); non-essential sugar transporter; pseudogene in *M. leprae*	(7,8)
Parent	*rv2931, pspA*	SNP: E419K; Phenolpthiocerol synthesis type-I polyketide synthase; non-essential for growth; present in *M. leprae*; multiple SNPs in *M. tuberculosis* strains: G549S, A803T, V862I, R877H, H955P	(7,12)

Supplementary References

1. Stapleton MR, Smith LJ, Hunt DM, Buxton RS and Green J. (2012) *Mycobacterium tuberculosis* WhiB1 represses transcription of the essential chaperonin GroEL2. *Tuberculosis (Edinb)*. **92**:328-332.

2. Cortes T, Schubert OT, Rose G, Arnvig KB, Comas I, Aebersold R and Young DB. (2013) Genome-wide mapping of transcriptional start sites defines an extensive leaderless transcriptome in *Mycobacterium tuberculosis*. *Cell Rep*. **5**:1121-1131.

3. Waddell SJ and Butcher PD. (2010) Use of DNA arrays to study transcriptional responses to antimycobacterial compounds. In *Antibiotic Resistance Protocols* (SH Gillespie and TD McHugh Eds.) *Methods Mol Biol.* **642**:75-91

4. Cheah ES, Malkin J, Free RC, Lee S-M, Perera N, Woltmann G, Patel H, Kimmitt PT, Smith RJ and Rajakumar K. (2010) A two-tube combined Taqman/SYBR Green assay to identify mycobacteria and detect single global lineage-defining polymorphisms in *Mycobacterium tuberculosis*. J Mol Diagn. **12**:250-256.

5. Snapper SB, Melton RE, Mustafa S, Kieser T and Jacobs W R. (1990) Isolation and characterization of efficient plasmid transformation mutants of *Mycobacterium smegmatis*. *Mol Microbiol.* **4:**1911–1919.

6. Parish T and Stoker NG. (2000) Use of a flexible cassette method to generate a double unmarked *Mycobacterium tuberculosis tlyA plcABC* mutant by gene replacement. *Microbiology* **146**:1969-1975.

7. Lew JM, Kapopoulou A, Jones LM and Cole ST. (2011) TubercuList - 10 years after. *Tuberculosis (Edinb.)* **91**:1-7.

8. Leprosy. http://mycobrowser.epfl.ch/leprosy.html

9. Griffin JE, Gawronski JD, Dejesus MA, Ioerger TR, Akerley BJ, Sassetti CM (2011) High-resolution phenotypic profiling defines genes essential for mycobacterial growth and cholesterol catabolism. *PLoS Pathog.* **7**:e1002251.

10. Tsolaki AG, Hirsh AE, DeRiemer K, Enciso JA, Wong MZ, Hannan M, Goguet de la Salmoniere YO, Aman K, Kato-Maeda M and Small PM. (2004) Functional and evolutionary genomics of *Mycobacterium tuberculosis*: insights from genomic deletions in 100 strains. *Proc Natl Acad Sci USA.* **101**:4865-4870.

11. Sureka K, Dey S, Datta P, Singh AK, Dasgupta A, Rodrigue S, Basu J and Kundu M. (2007) Polyphosphate kinase is involved in stress-induced *mprAB-sigE-rel* signalling in mycobacteria. *Mol Microbiol* . **65**: 261-276.

12. Lamichhane G, Zignol M, Blades NJ, Geiman DE, Dougherty A, Grosset J, Broman KW and Bishai WR. (2003) A postgenomic method for predicting essential genes at subsaturation levels of mutagenesis: application to *Mycobacterium tuberculosis*. *Proc Natl Acad Sci USA*. **100**:7213-7218.
